# Metabolic Changes in Skin Caused by *Scd1* Deficiency: A Focus on Retinol Metabolism

**DOI:** 10.1371/journal.pone.0019734

**Published:** 2011-05-09

**Authors:** Matthew T. Flowers, Chad M. Paton, Sheila M. O'Byrne, Kevin Schiesser, John A. Dawson, William S. Blaner, Christina Kendziorski, James M. Ntambi

**Affiliations:** 1 Department of Biochemistry, University of Wisconsin-Madison, Madison, Wisconsin, United States of America; 2 Department of Nutritional Sciences, University of Wisconsin-Madison, Madison, Wisconsin, United States of America; 3 Division of Animal and Nutritional Sciences, West Virginia University, Morgantown, West Virginia, United States of America; 4 Institute of Human Nutrition and Department of Medicine, Columbia University, New York, New York, United States of America; 5 Department of Biostatistics and Medical Informatics, University of Wisconsin-Madison, Madison, Wisconsin, United States of America; Hospital Universitario 12 de Octubre, Spain

## Abstract

We previously reported that mice with skin-specific deletion of stearoyl-CoA desaturase-1 (*Scd1*) recapitulated the skin phenotype and hypermetabolism observed in mice with a whole-body deletion of *Scd1*. In this study, we first performed a diet-induced obesity experiment at thermoneutral temperature (33°C) and found that skin-specific *Scd1* knockout (SKO) mice still remain resistant to obesity. To elucidate the metabolic changes in the skin that contribute to the obesity resistance and skin phenotype, we performed microarray analysis of skin gene expression in male SKO and control mice fed a standard rodent diet. We identified an extraordinary number of differentially expressed genes that support the previously documented histological observations of sebaceous gland hypoplasia, inflammation and epidermal hyperplasia in SKO mice. Additionally, transcript levels were reduced in skin of SKO mice for genes involved in fatty acid synthesis, elongation and desaturation, which may be attributed to decreased abundance of key transcription factors including SREBP1c, ChREBP and LXRα. Conversely, genes involved in cholesterol synthesis were increased, suggesting an imbalance between skin fatty acid and cholesterol synthesis. Unexpectedly, we observed a robust elevation in skin retinol, retinoic acid and retinoic acid-induced genes in SKO mice. Furthermore, SEB-1 sebocytes treated with retinol and SCD inhibitor also display an elevation in retinoic acid-induced genes. These results highlight the importance of monounsaturated fatty acid synthesis for maintaining retinol homeostasis and point to disturbed retinol metabolism as a novel contributor to the *Scd1* deficiency-induced skin phenotype.

## Introduction

The epidermis has a large capacity for synthesizing both fatty acids and cholesterol, which are utilized to form a variety of complex lipids including phospholipids, triglycerides, sphingolipids, esterified cholesterol, wax esters and retinyl esters [1], [2], [3]. These epidermal lipids are essential for maintaining a permeability barrier that protects against transepidermal loss of water and electrolytes, as well as providing an anti-microbial barrier that prevents microorganism colonization and infection [1], [3], [4]. Disruption of the epidermal barrier stimulates both sterol and fatty acid synthesis, an adaptation that aids in the restoration of normal barrier function [3].

Skin is a stratified tissue composed of the epidermis, dermis and subcutaneous fat layers. The epidermis is the thinnest of the three layers, but mitotically is the most active layer due to the continuous differentiation of keratinocytes into the cornified epithelium, which is exposed to the environment. The dermis is thicker than the epidermis and is composed largely of fibroblasts, which surround the vasculature, nerves, immune cells, hair follicle and the attached sebaceous gland. The major function of sebaceous gland is to release lipid complex-lubricants, termed sebum, into the sebaceous duct and hair follicle via rupture of differentiated sebocytes [2]. However, the sebaceous gland has also been proposed to be involved in antioxidant and antibacterial effects, pheromone transport and epidermal hydration [2], [5]. Sebum contains triglycerides, diglycerides, fatty acids, cholesterol, cholesteryl esters, squalene and wax esters [2]. Whereas overproduction of sebum by the sebaceous gland contributes to the development of acne and seborrhea, inadequate sebum production due to sebocyte dysfunction impairs the function of the hair follicle [2].

SCD1 is highly expressed in the sebaceous gland and is not observed in the hair follicle or any other cell type in mouse skin [6]. Mice with a whole-body or skin-specific deletion of *Scd1* develop severe sebaceous gland hypoplasia that results in progressive scarring alopecia, indicating that SCD1 is critical for normal sebaceous gland function [6], [7], [8], [9]. SCD1 is a Δ9 fatty acid desaturase that primarily catalyzes the conversion of the saturated fatty acids palmitic acid (16∶0) and stearic acid (18∶0) into the *cis*-monounsaturated fatty acids (MUFA) palmitoleic acid (16∶1n7) and oleic acid (18∶1n9), respectively [10]. The MUFA serve as important esterification substrates in the formation of triglycerides, cholesterol esters and wax esters, which are components of the sebum [7], [11]. Both whole-body and skin-specific deletion of *Scd1* cause a remarkable hypermetabolic phenotype that protects against the development of both genetic- and diet-induced obesity, fatty liver and insulin resistance [7], [8], [12], [13], [14], [15], [16]. These metabolic phenotypes persist despite hyperphagia, suggesting that their obesity resistance is derived from an increase in energy expenditure. *Scd1*-deficient mice are also cold intolerant, suggesting that they have increased cold perception and/or loss of heat to the environment [7], [13], [17]. The increased energy expenditure in these mice has been hypothesized to be due to upregulation of thermogenic processes for temperature maintenance at the expense of fuel economy [7], [13]. However, the exact mechanism by which reduced sebaceous gland MUFA synthesis in the skin elicits signals to cause hypermetabolism has not been determined.

In the current study, we performed microarray analysis of skin gene expression using Affymetrix 430 2.0 arrays to explore the cellular mechanisms responsible for the sebaceous gland hypoplasia and associated skin phenotypes in mice with skin-specific deletion of *Scd1* (SKO mice). The gene expression profile supports the previous histological observations of sebaceous gland hypoplasia, inflammation, hyperkeratosis, epidermal hyperplasia and tissue remodeling [18]. Additionally, the gene expression pattern suggests an imbalance in skin lipogenesis characterized by increased sterol synthesis but decreased fatty acid synthesis and alterations in fatty acid composition. Unexpectedly, retinoic acid-responsive genes, as well as skin levels of retinol and retinoic acid, were remarkably elevated. These results support a novel and important role for skin MUFA synthesis in maintaining cellular retinol homeostasis and suggest that the origin of the skin phenotype in SKO mice is due to severe retinoic acid-induced sebaceous gland hypoplasia.

## Results

### Thermoneutral environment does not rescue obesity resistance

**Figure 1:**
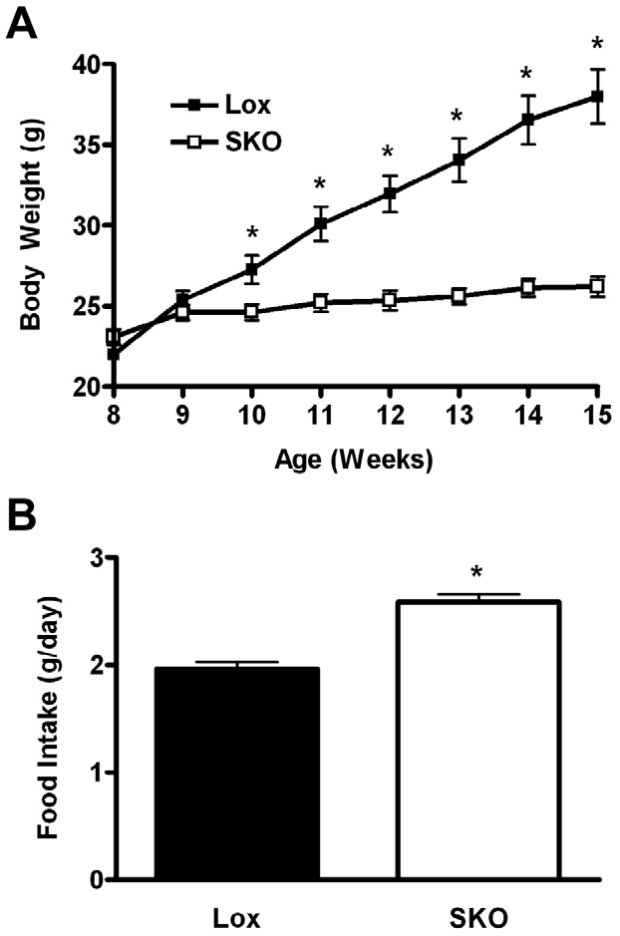
SKO mice remain resistant to obesity in a thermoneutral environment. Male Lox and SKO mice (n = 9–10) were transferred at 6 weeks of age to a controlled environment (32.5–33.5°C; 30–40% relative humidity), allowed to acclimate for 2 weeks and then fed a high-fat diet (Harlan TD.06414; 60% kcal fat) for 7 weeks. A) Body weight curve; B) Food intake. Data represent mean ± SEM. *, *p*<0.05.

We previously reported that SKO mice recapitulate the hypermetabolic phenotype observed in mice with a whole-body deletion of SCD1, resulting in resistance to high-fat diet-induced obesity when housed at ambient temperature (21°C) [7]. To investigate whether loss of heat to the environment contributes to the obesity protection, we repeated the diet-induced obesity experiment in SKO mice at thermoneutral temperature (32.5–33.5°C). Mice were fed a high-fat diet (60% kcal fat; Harlan TD.06414) for 7 weeks at this temperature. Surprisingly, the obesity resistance phenotype and hyperphagia persisted even at the elevated temperature (**Figure 1**), similar to our previous observations in high-fat fed SKO housed at ambient temperature [7].

### Microarray analysis of skin of SKO mice

To explore mechanisms other than heat loss that may be responsible for the skin-derived alteration in whole-body energy metabolism in SKO mice, we analyzed skin gene expression in 8–9 week old male SKO mice (n = 3) and *Scd1^flox/flox^* (Lox) control mice (n = 3) using Affymetrix 430 2.0 microarrays. The summary of the number of probe sets found to be differentially expressed by either EBarrays or Welch's t-test are shown in **Table 1**. Applying EBarrays with the posterior probability of differential expression (DE) set at 0.639 (soft 5% false discovery rate (FDR) cutoff) or 0.95 (hard 5% FDR cutoff) yielded 8648 and 6433 DE probe sets, respectively. The Welch's t-test yielded 969 DE probe sets with q-values less than 0.05. When comparing the two approaches, 897 of those 969 probe sets identified as DE via t-tests are also identified as DE by EBarrays (posterior probability of DE >0.95). The set of transcripts differentially expressed between Lox and SKO is enriched for 16 gene ontology (GO) terms, which are listed in **Table 2**.

**Table 1:** Summary of Differentially Expressed Probe Sets (DEPS).

Method	Total DEPS	Increased	Decreased
EBarrays (soft); 5% FDR PP of DE>0.639	8648	4558(1495)	4090(1313)
EBarrays (hard); 5% FDR PP of DE>0.95	6433	3415(1469)	3018(1276)
Welch's t-test (q<0.05)	969	550(377)	419(236)
Welch's t-test AND EBarrays Intersect	897	516(377)	381(236)

Affymetrix Mouse 430 2.0 microarray chips were used to search for skin gene expression changes between male Lox and SKO mice. Lists of differentially expressed probe sets (DEPS) were generated using Welch's t-tests or EBarrays, as described in *Methods*. For each comparison, the number of total, increased and decreased DEPS is shown, and those with a fold-change of 2-fold or more are indicated in parentheses.

**Table 2:** Enriched Gene Ontology (GO) Terms.

Myofibril	Contractile Fiber	Sacromere	Contractile Fiber Part
Actin Binding	Actin Cytoskeleton	Cytoskeletal Protein Binding	I Band
Anatomical Structure Formation Involved in Morphogenesis	Z Disc	Cell-cell Junction	Ectoderm Development
Epidermis Development	Skeletal System Development	Apicolateral Plasma Membrane	Muscle System Process

Tests of enrichment of GO terms via overrepresentation were conducted on the set of 8648 differentially expressed probe sets identified by EBarrays. The tests were performed using the allez R package. GO terms were said to be overrepresented if they had at least 5 distinct probe sets in the differentially expressed list being analyzed, but no more than 500, and surpassed a Z-score cutoff of 5. GO terms are listed in left-to-right and top-to-bottom decreasing order of significance. The same set of criteria was used to assess enrichment using KEGG pathways, but no such pathway was found for this dataset.

### Sebaceous gland hypoplasia, epidermal hyperplasia and inflammation

Studies by Sundberg *et al.* in the naturally occurring *Scd1^ab-2j^* mutant have suggested that SCD1 is required for a functional sebaceous gland and for the degradation of the inner root sheath from the emerging hair shaft [8]. Thus, MUFA production by the sebaceous gland is critical for sebocyte maintenance and function. Consistent with the previous histological observation of sebaceous gland hypoplasia [19], [20], we observed decreased expression in SKO skin of two key markers of terminal sebocyte differentiation, *Scd3* and *Mc5R* (**Table 3**). The expression of the cytochrome P450 1A1 (*Cyp1a1*), which encodes a xenobiotic metabolizing enzyme expressed in the sebaceous gland [21], was also largely ablated (**Table 3**). Additionally, the expression of androgen receptor (*Ar*), which is enriched in the basal layer of the sebaceous gland and in outer root sheath keratinocytes of the hair follicle [22], was dramatically suppressed in skin of SKO mice (**Table 3**).

**Table 3:** Sebaceous gland or sebocyte markers.

AFFY ID	Gene Symbol	Gene Name	FC	PP of DE	q-value
1460723_at	*Mc5r*	melanocortin 5 receptor	**0.59**	0.99	0.111
1437064_at	*Ar*	androgen receptor	**0.31**	1.00	0.153
1455647_at	*Ar*	androgen receptor	**0.22**	1.00	0.107
1422217_a_at	*Cyp1a1*	cytochrome P450, family 1, subfamily a, polypeptide 1	**0.19**	1.00	0.023
1450956_at	*Scd3*	stearoyl-CoA desaturase 3	**0.09**	1.00	0.052
1423366_at	*Scd3*	stearoyl-CoA desaturase 3	**0.08**	1.00	0.053

Changes in gene expression are reported as fold-change (FC) relative to Lox mice. Significant differences between Lox and SKO were determined as described in *Methods*, and for both Welch's t-test and EBarrays the false discovery rate was set at 5%. All probe sets listed have a posterior probability of differential expression (PP of DE) >0.639 (soft threshold) based upon analysis by EBarrays. Additionally, Welch's t-test was used to calculate q-values and those probe sets with q-values<0.05 were considered significant.

The impaired degradation of the inner root sheath due to *Scd1* deficiency-related sebaceous gland dysfunction is hypothesized to cause restraint and destruction of the hair follicle, inducing an inflammatory reaction, epidermal hyperplasia and scarring alopecia [8]. Consistent with these observations, we observed increased expression of several transcripts supporting prominent keratinocyte activation and differentiation including keratin 6, 16 and 17 (*Krt6a*, *Krt6b*, *Krt16*, *Krt17*), involucrin (*Ivl*), filaggrin (*Flg)*, transglutaminase 1 (*Tgm1*), and several genes encoding members of the small proline-rich and late cornified envelope protein family (**Table 4**).

**Table 4:** Keratinocyte differentiation and activation; epidermal barrier formation.

AFFY ID	Gene Symbol	Gene Name	FC	PP of DE	q-value
1422672_at	*Sprr1b*	small proline-rich protein 1B	**29.95**	1.00	0.114
1456248_at	*Lce3f*	late cornified envelope 3F	**20.40**	1.00	0.083
1422240_s_at	*Sprr2h*	small proline-rich protein 2H	**17.52**	1.00	0.075
1448932_at	*Krt16*	keratin 16	**16.26**	1.00	0.101
1439016_x_at	*Sprr2a*	small proline-rich protein 2A	**12.27**	1.00	0.044
1450618_a_at	*Sprr2a*	small proline-rich protein 2A	**11.67**	1.00	0.045
1422588_at	*Krt6b*	keratin 6B	**11.01**	1.00	0.165
1422783_a_at	*Krt6a*	keratin 6A	**10.57**	1.00	0.023
1449133_at	*Sprr1a*	small proline-rich protein 1A	**7.82**	1.00	0.04
1427700_x_at	*Krt6a*	keratin 6A	**7.35**	1.00	0.037
1420771_at	*Sprr2d*	small proline-rich protein 2D	**7.20**	0.99	0.172
1420193_at	*Krt17*	keratin 17	**6.62**	1.00	0.037
1456001_at	*Lce3a*	late cornified envelope 3A	**6.51**	1.00	0.157
1422963_at	*Sprr2i*	small proline-rich protein 2I	**6.42**	0.87	0.225
1422784_at	*Krt6a*	keratin 6A	**5.34**	1.00	0.019
1419317_x_at	*Lce3c*	late cornified envelope 3C	**4.81**	1.00	0.156
1427268_at	*Flg*	Filaggrin	**4.68**	1.00	0.072
1421316_at	*Lce1g*	late cornified envelope 1G	**4.63**	1.00	0.1
1429540_at	*Cnfn*	Cornifelin	**2.93**	1.00	0.066
1423227_at	*Krt17*	keratin 17	**2.88**	1.00	0.03
1451416_a_at	*Tgm1*	Transglutaminase 1, K polypeptide	**2.58**	1.00	0.073
1439878_at	*Ivl*	Involucrin	**2.50**	1.00	0.107
1422222_at	*Ivl*	Involucrin	**1.87**	1.00	0.1
1420550_at	*Lce1f*	late cornified envelope 1F	**1.84**	1.00	0.052
1418855_at	*Lce1l*	late cornified envelope 1L	**0.66**	0.94	0.132
1429565_s_at	*Lce1m*	late cornified envelope 1M	**0.33**	1.00	0.042

See Table 3 for legend.

The skin phenotype due to *Scd1* deficiency is consistent with atopic dermatitis, involving inflammation and pruritis. Although this often culminates in spontaneous ulcerative dermatisis in older mice (unpublished observations), none of the mice used in the current study displayed this condition. Skin inflammation is evident from the increased expression of a large number of chemokine ligands, chemokine receptors and other mediators of the inflammatory process including transforming growth factor beta (*Tgfb1*), tumor necrosis factor (*Tnf*), several members of the NF-kb pathway and others listed in **Table 5** and **Table S1**. Several members of the interleukin family of cytokines were elevated including interleukin (IL)-1β (*Il1b*), IL-18 (*Il18*), IL-33 (*Il33*), and IL1-family members 5, 6, 8 and 9 (*Il1f5*, *Il1f6*, *Il1f8*, *Il1f9*). We also observed a modest increase in the expression level of the receptors for IL-1 and IL-18 (*Il1r1* and *Il18r1*), and the IL-1 accessory protein (*Il1rap*). Activation of the IL-1 pathway may be further influenced by the observed increase in expression of the IL-1 receptor antagonist (*Il1rn*) and decrease in the expression of the IL-1 decoy receptor *Il1r2*, which decreases the activity of the ligands for the active IL-1 receptor *Il1r1*. Notably, we observed a large increase in *Cd44*, which increases with inflammation and modulates epidermal proliferation and inflammatory responses [23]. Inflammation can also be influenced by fatty acid products of lipoxygenases. We observed ∼2-fold decrease in the expression of platelet-type 12-lipoxygenase (*Alox12*), which has previously been shown to be important for a normal permeability barrier [24], as well as decreased expression of 5-lipoxygenase *Alox5* (**Table S2**). However, expression of the epidermal-type 12-lipoxygenase *Alox12e* and the leukocyte-type 12-lipoxygenase *Alox15* were increased 2.5 to 3-fold.

**Table 5:** Inflammation, wound healing and defense response.

AFFY ID	Gene Symbol	Gene Name	FC	PP of DE	q-value
1438148_at	*Cxcl3*	chemokine (C-X-C motif) ligand 3	**30.63**	1.00	0.11
1450826_a_at	*Saa3*	serum amyloid A 3	**25.47**	1.00	0.055
1449984_at	*Cxcl2*	chemokine (C-X-C motif) ligand 2	**22.85**	1.00	0.075
1448377_at	*Slpi*	secretory leukocyte peptidase inhibitor	**21.69**	1.00	0.057
1448756_at	*S100a9*	S100 calcium binding protein A9 (calgranulin B)	**20.77**	1.00	0.058
1450788_at	*Saa1*	serum amyloid A 1	**14.12**	1.00	0.055
1419394_s_at	*S100a8*	S100 calcium binding protein A8 (calgranulin A)	**11.98**	1.00	0.055
1419728_at	*Cxcl5*	chemokine (C-X-C motif) ligand 5	**8.98**	1.00	0.102
1418609_at	*Il1f6*	interleukin 1 family, member 6	**7.85**	1.00	0.082
1419209_at	*Cxcl1*	chemokine (C-X-C motif) ligand 1	**7.71**	1.00	0.089
1421688_a_at	*Ccl1*	chemokine (C-C motif) ligand 1	**6.36**	1.00	0.061
1419492_s_at	*Defb1*	defensin beta 1	**5.40**	1.00	0.06
1422029_at	*Ccl20*	chemokine (C-C motif) ligand 20	**5.00**	1.00	0.089
1419491_at	*Defb1*	defensin beta 1	**4.68**	1.00	0.062
1419600_at	*Defb4*	defensin beta 4	**4.66**	1.00	0.053
1426300_at	*Alcam*	activated leukocyte cell adhesion molecule	**4.35**	1.00	0.044
1419561_at	*Ccl3*	chemokine (C-C motif) ligand 3	**4.10**	1.00	0.122
1417932_at	*Il18*	interleukin 18	**4.04**	1.00	0.039
1421806_at	*Defb3*	defensin beta 3	**3.97**	1.00	0.031
1449399_a_at	*Il1b*	interleukin 1 beta	**3.89**	1.00	0.082
1434376_at	*Cd44*	CD44 antigen	**3.73**	1.00	0.06
1452483_a_at	*Cd44*	CD44 antigen	**3.68**	1.00	0.071
1423760_at	*Cd44*	CD44 antigen	**3.65**	1.00	0.099
1448823_at	*Cxcl12*	chemokine (C-X-C motif) ligand 12	**3.48**	1.00	0.068
1417925_at	*Ccl22*	chemokine (C-C motif) ligand 22	**3.28**	1.00	0.052
1437467_at	*Alcam*	activated leukocyte cell adhesion molecule	**3.26**	1.00	0.058
1417574_at	*Cxcl12*	chemokine (C-X-C motif) ligand 12	**3.20**	1.00	0.039
1418457_at	*Cxcl14*	chemokine (C-X-C motif) ligand 14	**3.11**	1.00	0.113
1449195_s_at	*Cxcl16*	chemokine (C-X-C motif) ligand 16	**2.99**	1.00	0.062
1417483_at	*Nfkbiz*	nuclear factor of kappa light polypeptide gene enhancer in B-cells inhibitor, zeta	**2.87**	1.00	0.082
1437466_at	*Alcam*	activated leukocyte cell adhesion molecule	**2.84**	1.00	0.04
1418718_at	*Cxcl16*	chemokine (C-X-C motif) ligand 16	**2.78**	1.00	0.067
1451798_at	*Il1rn*	interleukin 1 receptor antagonist	**2.77**	1.00	0.051
1426301_at	*Alcam*	activated leukocyte cell adhesion molecule	**2.74**	1.00	0.073
1423466_at	*Ccr7*	chemokine (C-C motif) receptor 7	**2.72**	1.00	0.069
1448710_at	*Cxcr4*	chemokine (C-X-C motif) receptor 4	**2.69**	1.00	0.129
1419609_at	*Ccr1*	chemokine (C-C motif) receptor 1	**2.58**	0.99	0.175
1418456_a_at	*Cxcl14*	chemokine (C-X-C motif) ligand 14	**2.48**	1.00	0.054
1421807_at	*Defb6*	defensin beta 6	**2.41**	1.00	0.071
1425715_at	*Il1f8*	interleukin 1 family, member 8	**2.34**	1.00	0.148
1433699_at	*Tnfaip3*	tumor necrosis factor, alpha-induced protein 3	**2.28**	1.00	0.037
1457644_s_at	*Cxcl1*	chemokine (C-X-C motif) ligand 1	**2.21**	1.00	0.104
1418424_at	*Tnfaip6*	tumor necrosis factor alpha induced protein 6	**2.19**	1.00	0.127
1419413_at	*Ccl17*	chemokine (C-C motif) ligand 17	**2.16**	1.00	0.077
1425663_at	*Il1rn*	interleukin 1 receptor antagonist	**2.12**	1.00	0.095
1421370_a_at	*Il1f5*	interleukin 1 family, member 5 (delta)	**2.04**	1.00	0.109

See Table S1 for additional probe sets. See Table 3 for legend.

Increased expression of genes encoding antimicrobial peptides of the defensin family (*Defb1*, *Defb3*, *Defb4* and *Defb6*) [25], injury-induced calcium-binding proteins (*S100a8* and *S100a9*) [26] and the acute phase response proteins serum amyloid A1–A3 (*Saa1*, *Saa2* and *Saa3*) are consistent with a response to tissue damage and inflammation (**Table 5** and **Table S1**). Some genes in this category were decreased, which may indicate an anti-inflammatory process or an adaptation to prolonged inflammation. Increased expression of the *Jun*, *Fos* and *Cebpb* transcription factors suggest activation of the inflammatory and proliferating phase of the wound healing process (**Table 6**). Several other transcriptional changes in SKO mice parallel other models of wound healing and dermatitis including elevated expression of tenascin C (*Tnc*) [27], osteopontin (*Spp1*) [28], thrombospondin-1 (*Thbs1*) [29] and *Socs3* [30] (**Table 7**).

**Table 6:** Transcription factors and co-activators.

AFFY ID	Gene Symbol	Gene Name	FC	PP of DE	q-value
1417065_at	*Egr1*	early growth response 1	**4.95**	1.00	0.031
1418175_at	*Vdr*	vitamin D receptor	**3.68**	1.00	0.032
1418176_at	*Vdr*	vitamin D receptor	**3.10**	1.00	0.057
1417981_at	*Insig2*	insulin induced gene 2	**3.09**	1.00	0.066
1417982_at	*Insig2*	insulin induced gene 2	**3.07**	1.00	0.048
1417980_a_at	*Insig2*	insulin induced gene 2	**2.90**	1.00	0.074
1423100_at	*Fos*	FBJ osteosarcoma oncogene	**2.88**	1.00	0.058
1435950_at	*Hr*	Hairless	**2.25**	1.00	0.019
1427844_a_at	*Cebpb*	CCAAT/enhancer binding protein (C/EBP), beta	**2.24**	1.00	0.082
1449945_at	*Ppargc1b*	peroxisome proliferative activated receptor, gamma, coactivator 1 beta	**2.07**	1.00	0.116
1417409_at	*Jun*	Jun oncogene	**2.07**	1.00	0.103
1448694_at	*Jun*	Jun oncogene	**2.04**	1.00	0.041
1422110_at	*Hr*	Hairless	**1.92**	1.00	0.097
1439797_at	*Ppard*	peroxisome proliferator activator receptor delta	**1.89**	1.00	0.07
1418901_at	*Cebpb*	CCAAT/enhancer binding protein (C/EBP), beta	**1.67**	1.00	0.036
1422631_at	*Ahr*	aryl-hydrocarbon receptor	**1.65**	0.82	0.18
1417395_at	*Klf4*	Kruppel-like factor 4 (gut)	**1.47**	0.96	0.078
1438796_at	*Nr4a3*	nuclear receptor 4a3	**1.46**	0.98	0.092
1457721_at	*Ppara*	peroxisome proliferator activated receptor alpha	**0.72**	0.94	0.13
1420715_a_at	*Pparg*	peroxisome proliferator activated receptor gamma	**0.70**	0.98	0.094
1437751_at	*Ppargc1a*	peroxisome proliferative activated receptor, gamma, coactivator 1 alpha	**0.70**	0.83	0.152
1448886_at	*Gata3*	GATA binding protein 3	**0.68**	0.99	0.074
1436325_at	*Rora*	RAR-related orphan receptor alpha	**0.59**	0.82	0.184
1426997_at	*Thra*	thyroid hormone receptor alpha	**0.58**	1.00	0.065
1416505_at	*Nr4a1*	nuclear receptor subfamily 4a1	**0.56**	0.91	0.163
1456395_at	*Ppargc1a*	peroxisome proliferative activated receptor, gamma, coactivator 1 alpha	**0.56**	1.00	0.082
1454734_at	*Lef1*	lymphoid enhancer binding factor 1	**0.56**	1.00	0.101
1426690_a_at	*Srebf1*	sterol regulatory element binding transcription factor 1	**0.53**	1.00	0.056
1454671_at	*Insig1*	insulin induced gene 1	**0.51**	0.96	0.159
1434099_at	*Ppargc1a*	peroxisome proliferative activated receptor, gamma, coactivator 1 alpha	**0.51**	1.00	0.141
1426464_at	*Nr1d1*	nuclear receptor subfamily 1d1	**0.51**	1.00	0.051
1450444_a_at	*Nr1h3*	nuclear receptor subfamily 1h3	**0.51**	1.00	0.132
1434100_x_at	*Ppargc1a*	peroxisome proliferative activated receptor, gamma, coactivator 1 alpha	**0.47**	1.00	0.152
1425792_a_at	*Rorc*	RAR-related orphan receptor gamma	**0.45**	1.00	0.058
1454675_at	*Thra*	thyroid hormone receptor alpha	**0.44**	1.00	0.054
1439675_at	*Ppara*	peroxisome proliferator activated receptor alpha	**0.44**	1.00	0.039
1457635_s_at	*Nr3c1*	nuclear receptor subfamily 3c1	**0.41**	1.00	0.114
1418782_at	*Rxrg*	Retinoid X receptor gamma	**0.38**	1.00	0.069
1419185_a_at	*Mlxipl*	MLX interacting protein-like	**0.30**	1.00	0.056

See Table 3 for legend.

**Table 7:** Increased in wounded and inflammed skin, psoriasis.

AFFY ID	Gene Symbol	Gene Name	FC	PP of DE	q-value
1416342_at	*Tnc*	Tenascin C	**26.95**	1.00	0.05
1449254_at	*Spp1*	secreted phosphoprotein 1	**24.88**	1.00	0.041
1460302_at	*Thbs1*	thrombospondin 1	**11.57**	1.00	0.019
1418350_at	*Hbegf*	Heparin-binding EGF-like growth factor	**10.39**	1.00	0.08
1418349_at	*Hbegf*	Heparin-binding EGF-like growth factor	**9.59**	1.00	0.037
1421943_at	*Tgfa*	Transforming growth factor alpha	**2.92**	1.00	0.053
1450271_at	*Ptk6*	PTK6 protein tyrosine kinase 6	**2.37**	1.00	0.123
1455899_x_at	*Socs3*	suppressor of cytokine signaling 3	**2.03**	1.00	0.07
1416576_at	*Socs3*	suppressor of cytokine signaling 3	**1.93**	1.00	0.086
1456212_x_at	*Socs3*	suppressor of cytokine signaling 3	**1.92**	1.00	0.084
1442923_at	*Ptk6*	PTK6 protein tyrosine kinase 6	**1.79**	0.96	0.158

See Table 3 for legend.

Increased tissue remodeling is supported by differential expression of a wide array of proteolytic genes encoding matrix metalloproteases, tissue-inhibitors of metalloproteases, cathepsins, and ADAM (a disintegrin and metalloproteinase) family proteins (**Table S3**). The differential expression of several genes encoding collagens, keratins, gap junction proteins, tight junction proteins and cadherins suggest rampant changes in keratinization, extracellular matrix composition and cell adhesion (**Table S4**). Of note, we observed a 38-fold increase in *Gjb2* (gap junction protein, beta 2; connexin-26), whose over-expression has been reported to keep epidermis in a hyperproliferative state, block the transistion to remodeling, lead to immune cell infiltration and delay epidermal barrier recovery [31]. Additionally, we observed increased expression of early growth response-1 (*Egr1*), which is important for TGF-β-dependent matrix remodeling [32] (**Table 6**).

### Imbalance of fatty acid and sterol synthesis

A proper balance of stratum corneum lipids is required for maintenance of normal barrier function. For example, mice topically treated with the sterol synthesis inhibitor lovastatin have decreased epidermal sterol synthesis but increased fatty acid synthesis coincident with the development of an impaired permeability barrier [33]. We have previously observed that hepatic *Scd1* deficiency blocks the dietary carbohydrate-induction of fatty acid synthesis and triglyceride accumulation [34]. Consistent with this pattern, the skin of SKO mice displayed decreased expression of mRNAs encoding key fatty acid synthesis enzymes including fatty acid synthase (*Fasn*), stearoyl-CoA desaturases-1 and -3 (*Scd1* and *Scd3*), elongases (*Elovl1*, *Elovl3*, *Elovl5 and Elovl6*) and SPOT14 (*Thrsp*), and triglyceride synthesis enzymes including *Gpam*, *Lpin1*, *Agpats* (-*2*, -*4*, and -*5*) and *Dgat1* (**Tables 8** and **9**). In contrast, we observed increased mRNA expression for the Δ9- and Δ6-desaturases *Scd2* and *Fads2*, respectively, along with the elongase *Elovl7*, which preferentially elongates saturated fatty acids [35] and the phosphatidic acid phosphatase *Lpin2*. Additionally, we observed decreased expression of several lipases including the triglyceride/diacylglyceride lipase *Pnpla2* (*Atgl*), diacylglyceride lipases *Dagla* and *Daglb* and the monoglyceride lipase *Mgll*, which is indicative of decreased hydrolysis of acylglycerol stores to free fatty acids and glycerol.

**Table 8:** Fatty acid synthesis, elongation and desaturation.

AFFY ID	Gene Symbol	Gene Name	FC	PP of DE	q-value
1418773_at	*Fads3*	fatty acid desaturase 3	**3.12**	1.00	0.055
1449219_at	*Fads3*	fatty acid desaturase 3	**3.09**	1.00	0.064
1435910_at	*Fads3*	fatty acid desaturase 3	**2.98**	1.00	0.07
1424097_at	*Elovl7*	ELOVL family member 7, elongation of long chain fatty acids (yeast)	**2.64**	1.00	0.096
1415823_at	*Scd2*	stearoyl-Coenzyme A desaturase 2	**2.51**	1.00	0.066
1440312_at	*Elovl7*	ELOVL family member 7, elongation of long chain fatty acids (yeast)	**1.78**	1.00	0.089
1441091_at	*Elovl7*	ELOVL family member 7, elongation of long chain fatty acids (yeast)	**1.71**	0.87	0.194
1419031_at	*Fads2*	fatty acid desaturase 2	**1.46**	0.82	0.152
1415822_at	*Scd2*	stearoyl-Coenzyme A desaturase 2	**1.40**	0.86	0.078
1436355_at	*Fads6*	fatty acid desaturase domain family, member 6	**0.69**	0.86	0.152
1443820_x_at	*Elovl1*	elongation of very long chain fatty acids (FEN1/Elo2, SUR4/Elo3, yeast)-like 1	**0.51**	1.00	0.105
1415964_at	*Scd1*	stearoyl-Coenzyme A desaturase 1	**0.47**	0.81	0.141
1443904_at	*Fads6*	fatty acid desaturase domain family, member 6	**0.42**	1.00	0.047
1420722_at	*Elovl3*	elongation of very long chain fatty acids (FEN1/Elo2, SUR4/Elo3, yeast)-like 3	**0.39**	1.00	0.069
1423828_at	*Fasn*	fatty acid synthase	**0.38**	1.00	0.071
1417404_at	*Elovl6*	ELOVL family member 6, elongation of long chain fatty acids (yeast)	**0.32**	1.00	0.095
1415965_at	*Scd1*	stearoyl-Coenzyme A desaturase 1	**0.25**	1.00	0.1
1415840_at	*Elovl5*	ELOVL family member 5, elongation of long chain fatty acids (yeast)	**0.20**	1.00	0.12
1437211_x_at	*Elovl5*	ELOVL family member 5, elongation of long chain fatty acids (yeast)	**0.19**	1.00	0.087
1424737_at	*Thrsp*	thyroid hormone responsive SPOT14 homolog (Rattus)	**0.16**	1.00	0.083
1422973_a_at	*Thrsp*	thyroid hormone responsive SPOT14 homolog (Rattus)	**0.14**	1.00	0.092
1450956_at	*Scd3*	stearoyl-CoA desaturase 3	**0.09**	1.00	0.052
1423366_at	*Scd3*	stearoyl-Coenzyme A desaturase 3	**0.08**	1.00	0.053

See Table 3 for legend.

**Table 9:** Acyltransferases, fatty acid binding proteins and lipases.

AFFY ID	Gene Symbol	Gene Name	FC	PP of DE	q-value
1416022_at	*Fabp5*	fatty acid binding protein 5, epidermal	**2.32**	1.00	0.093
1452836_at	*Lpin2*	lipin 2	**2.25**	1.00	0.075
1452837_at	*Lpin2*	lipin 2	**1.55**	1.00	0.112
1455448_at	*Dagla*	diacylglycerol lipase, alpha	**0.75**	0.68	0.113
1445229_at	*Dgat1*	diacylglycerol O-acyltransferase 1	**0.74**	0.83	0.095
1431331_at	*Mgll*	monoglyceride lipase	**0.73**	0.87	0.126
1434287_at	*Agpat5*	1-acylglycerol-3-phosphate O-acyltransferase 5 (lysophosphatidic acid acyltransferase, epsilon)	**0.69**	0.76	0.127
1428336_at	*Agpat4*	1-acylglycerol-3-phosphate O-acyltransferase 4 (lysophosphatidic acid acyltransferase, delta)	**0.57**	1.00	0.052
1419499_at	*Gpam*	glycerol-3-phosphate acyltransferase, mitochondrial	**0.56**	0.97	0.112
1426516_a_at	*Lpin1*	lipin 1	**0.56**	1.00	0.101
1451970_at	*Daglb*	diacylglycerol lipase, beta	**0.53**	0.93	0.133
1418288_at	*Lpin1*	lipin 1	**0.51**	1.00	0.069
1436640_x_at	*Agpat4*	1-acylglycerol-3-phosphate O-acyltransferase 4 (lysophosphatidic acid acyltransferase, delta)	**0.50**	1.00	0.096
1428143_a_at	*Pnpla2*	patatin-like phospholipase domain containing 2	**0.48**	1.00	0.05
1418295_s_at	*Dgat1*	diacylglycerol O-acyltransferase 1	**0.47**	1.00	0.107
1426785_s_at	*Mgll*	monoglyceride lipase	**0.44**	1.00	0.072
1450391_a_at	*Mgll*	monoglyceride lipase	**0.43**	1.00	0.067
1428821_at	*Agpat2*	1-acylglycerol-3-phosphate O-acyltransferase 2 (lysophosphatidic acid acyltransferase, beta)	**0.36**	1.00	0.117
1416023_at	*Fabp3*	fatty acid binding protein 3, muscle and heart	**0.19**	1.00	0.043

See Table 3 for legend.

As opposed to decreased fatty acid synthesis, key cholesterol synthesis genes such as HMG-CoA synthase-1 (*Hmgcs1*), HMG-CoA reductase (*Hmgcr*), 3-β-hydroxysterol-Δ24 reductase (*Dhcr24*), sterol-C5-desaturase (*Sc5d*) and squalene epoxidase (*Sqle*) were increased, while mevalonate decarboxylase (*Mvd*) was decreased (**Table 10**). The expression of the cholesterol sulfotransferase *Sult2b1*, which increases with keratinocyte differentiation and whose cholesterol sulfate product inhibits serine protease activity to prevent corneodesmosome degradation, was increased in SKO mice [36]. Overall, this gene expression pattern suggests that skin lipid synthesis is imbalanced in SKO mice, characterized by increased sterol synthesis and decreased fatty acid synthesis.

**Table 10:** Cholesterol synthesis.

AFFY ID	Gene Symbol	Gene Name	FC	PP of DE	q-value
1415993_at	*Sqle*	squalene epoxidase	**2.38**	1.00	0.083
1451457_at	*Sc5d*	sterol-C5-desaturase (fungal ERG3, delta-5-desaturase) homolog (S. cerevisae)	**2.33**	1.00	0.061
1427229_at	*Hmgcr*	3-hydroxy-3-methylglutaryl-Coenzyme A reductase	**2.18**	1.00	0.094
1418129_at	*Dhcr24*	24-dehydrocholesterol reductase	**2.15**	1.00	0.105
1417335_at	*Sult2b1*	sulfotransferase family, cytosolic, 2B, member 1	**1.93**	1.00	0.099
1433446_at	*Hmgcs1*	3-hydroxy-3-methylglutaryl-Coenzyme A synthase 1	**1.81**	1.00	0.102
1448663_s_at	*Mvd*	mevalonate (diphospho) decarboxylase	**0.57**	0.97	0.115

See Table 3 for legend.

Besides fatty acids and cholesterol, sphingolipids are important skin lipids for barrier acquisition. All three serine palmitoyltransferase genes (*Sptlc1–3*) were increased, suggesting increased sphingosine formation for ceramide synthesis (**Table 11**). We observed decreased expression of the sphingosine kinase (*Sphk2*) and increased expression of two sphingosine-1-phosphate phosphatases (*Sgpp1* and *Sgpp2*) as well as sphignosine phosphate lyase 1 (*Sgpl1*), suggestive of decreased levels of sphingosine-1-phosphate. Additionally, we observed decreased expression of the ceramide synthases *Lass4* and *Lass5* but increased expression of the dihydroceramide desaturases *Degs1* and *Degs2*. Most epidermal fatty acyl residues in ceramides are ≥C28 chain lenth and skin expresses five different ceramide synthase genes with different fatty acyl-CoA chain length preferences; however, it is currently not known which of the *Lass* gene products catalyzes the ≥C28 acylation of ceramide [37]. Glucosylceramide synthase (*Ugcg*), whose product glucosylceramides are the dominant epidermal glycosphingolipids and required for epidermal barrier function, was increased [38]. However, the expression of the fatty acid 2-hydroxylase *Fa2h* was decreased, indicative of decreased 2-OH ceramide and 2-OH glucosylceramide formation [39]. Additionally, we observed increased expression of sphingomyelin synthase 2 (*Sgms2*) and decreased expression of sphingomyelin phosphodiesterase 1 (*Smpd1*), suggesting increased utilization of ceramide for sphingomyelin synthesis. Overall, the sphingolipid and fatty acid gene expression pattern suggests that the machinery for ceramide and glucosylceramide is intact or upregulated, but that the composition of the fatty acyl chains is greatly altered.

**Table 11:** Sphingolipid synthesis.

AFFY ID	Gene Symbol	Gene Name	FC	PP of DE	q-value
1424549_at	*Degs2*	degenerative spermatocyte homolog 2 (Drosophila), lipid desaturase	**13.53**	1.00	0.053
1457867_at	*Sgpp2*	sphingosine-1-phosphate phosphotase 2	**7.35**	1.00	0.042
1415893_at	*Sgpl1*	sphingosine phosphate lyase 1	**2.51**	1.00	0.042
1421268_at	*Ugcg*	UDP-glucose ceramide glucosyltransferase	**2.41**	1.00	0.055
1421269_at	*Ugcg*	UDP-glucose ceramide glucosyltransferase	**2.41**	1.00	0.087
1435133_at	*Ugcg*	UDP-glucose ceramide glucosyltransferase	**2.23**	1.00	0.066
1422690_at	*Sptlc1*	serine palmitoyltransferase, long chain base subunit 1	**2.03**	1.00	0.052
1450015_x_at	*Sgpp1*	sphingosine-1-phosphate phosphatase 1	**1.89**	0.99	0.154
1436726_s_at	*Sptlc1*	serine palmitoyltransferase, long chain base subunit 1	**1.80**	1.00	0.065
1423345_at	*Degs1*	degenerative spermatocyte homolog 1 (Drosophila)	**1.77**	1.00	0.067
1415892_at	*Sgpl1*	sphingosine phosphate lyase 1	**1.64**	1.00	0.023
1429029_at	*Sgms2*	sphingomyelin synthase 2	**1.63**	1.00	0.044
1460243_at	*Sptlc2*	serine palmitoyltransferase, long chain base subunit 2	**1.48**	1.00	0.058
1439492_at	*Sptlc3*	serine palmitoyltransferase, long chain base subunit 3	**1.42**	0.93	0.091
1447874_x_at	*Smpd1*	sphingomyelin phosphodiesterase 1, acid lysosomal	**0.67**	1.00	0.082
1448621_a_at	*Smpd1*	sphingomyelin phosphodiesterase 1, acid lysosomal	**0.62**	1.00	0.07
1426230_at	*Sphk2*	sphingosine kinase 2	**0.61**	1.00	0.04
1447308_at	*Lass5*	LAG1 homolog, ceramide synthase 5	**0.45**	1.00	0.114
1451209_at	*Lass5*	LAG1 homolog, ceramide synthase 5	**0.37**	1.00	0.091
1417781_at	*Lass4*	LAG1 homolog, ceramide synthase 4	**0.36**	1.00	0.047
1417780_at	*Lass4*	LAG1 homolog, ceramide synthase 4	**0.31**	1.00	0.089
1426960_a_at	*Fa2h*	fatty acid 2-hydroxylase	**0.24**	1.00	0.052

See Table 3 for legend.

### Altered abundance of key metabolic transcription factors

The suppression of fatty acid synthesis may possibly be explained by the dramatic downregulation of several transcription factors involved in lipid metabolism (**Table 6**). We observed decreased expression of SREBP-1 (*Srebf1*), LXR-α (*Nr1h3*), ChREBP (*Mlxipl*), peroxisome proliferators activated receptor (PPAR) α and γ (*Ppara* and *Pparg*). Additionally, elevated expression of *Insig2* suggests a decrease in the maturation of SREBP-1 (**Table 6**). Despite the increased expression of several genes involved in cholesterol synthesis, the expression of *Srebp2* was not different. We also observed increased expression of PPARδ (*Ppard*) and PPARγ-coactivator 1β (*Ppargc1b*) (**Table 6**). This expression pattern is consistent with human keratinocytes treated with cytokines and UV light, in which the expression of *PPARD* is increased, but the expression of *PPARA*, *PPARG* and *NR1H3* was decreased [40]. In lesional skin of patients with psoriasis and atopic dermatitis the expression of *PPARD* was also increased while the expression of *PPARA* and *PPARG* was decreased [40], [41]. Although *Ppara* and one of its co-activators *Ppargc1a* were decreased in skin of SKO mice, we also observed increased expression of the fatty acid oxidation genes *Cpt1a*, *Acox1* and *Crot*, potentially due to the elevated expression of *Ppard* (**Table 6** and **Table S2**).

Several transcription factors important for skin development were also differentially expressed (**Table 6**). The expression of Kruppel-like factor 4 (*Klf4*), which is highly expressed in the differentiating layers of epidermis and is essential for barrier acquisition [42], is increased in SKO mice. Additionally, we observed decreased expression of *Gata3* and *Lef1*, which are important transcription factors involved in hair follicle and inner root sheath skin cell lineage [43]. Interestingly, expression of the vitamin D receptor (*Vdr*) and the nuclear receptor co-repressor hairless (*Hr*) were highly elevated in SKO mice. Hairless binds VDR to cause transcriptional repression [44], [45]. Overexpression of hairless in skin has been shown to cause delayed sebaceous gland differentiation, and an increased number of undifferentiated but decreased number of terminally differentiated keratinocytes [44], [46]. Several other metabolic transcription factors were also differentially expressed between Lox and SKO mice and are listed in **Table 6**.

### Accumulation of retinol and retinoic acid and activation of retinoic acid receptor (RAR)-target genes

**Figure 2:**
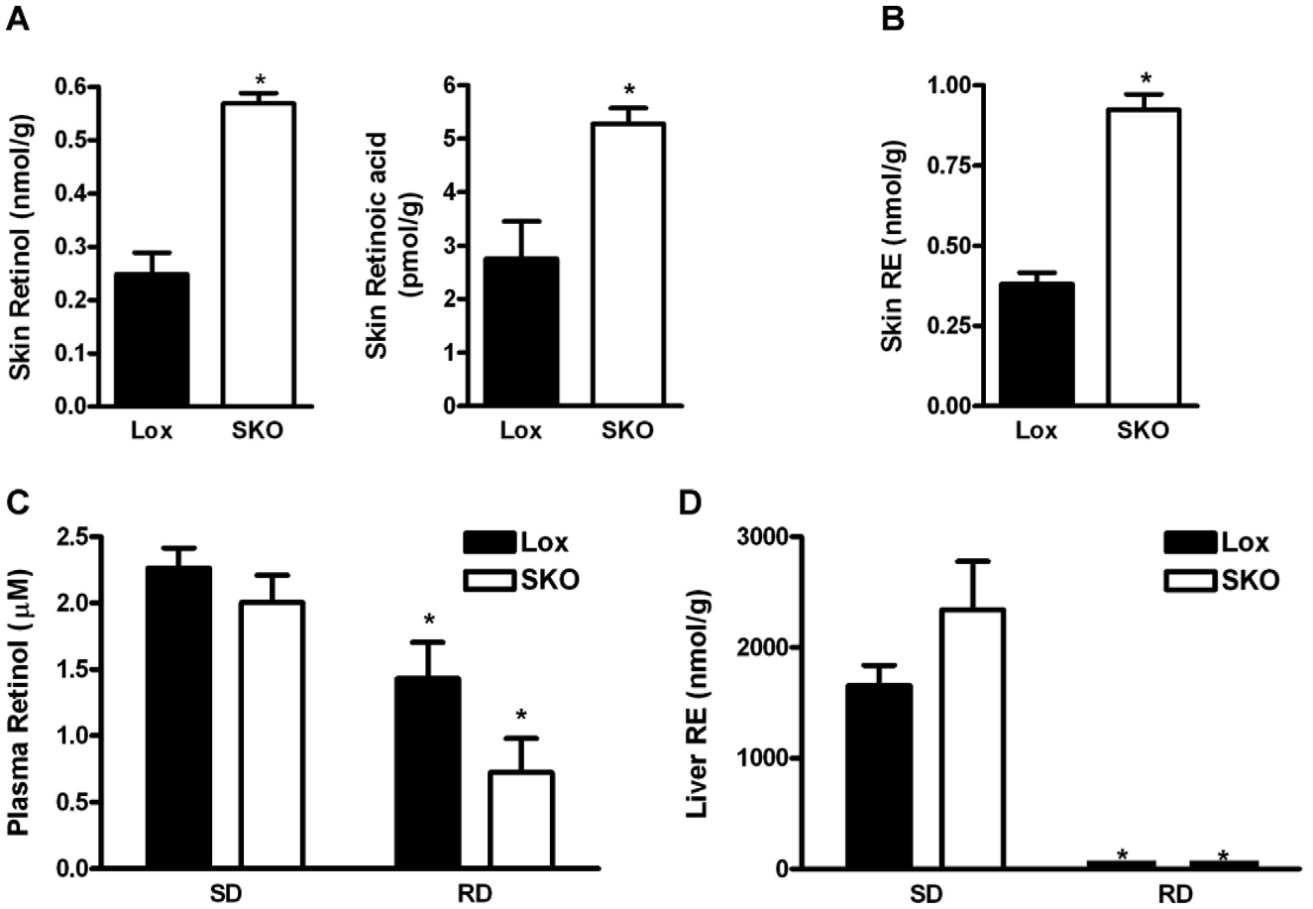
Altered retinoid homeostasis in SKO mice. A) Skin retinol and retinoic acid, as well as B) skin retinyl esters (RE) in male mice fed the standard diet (SD). * = *p*<0.05 for lox *vs.* SKO, n = 7–8 per group. C) Plasma retinol levels and D) liver RE levels in male mice fed either SD (n = 7–8 per group) or a retinoid-deficient (RD) diet (n = 6 per group). Data represent mean ± SEM. # = *p*<0.05 for SD *vs.* RD within same genotype.

One of the major metabolic pathways affected by the absence of skin SCD1 is retinol metabolism (**Table 12**). The genes encoding cellular retinol-binding protein-1 (*Rbp1*) and cellular retinoic acid-binding protein-2 (*Crabp2*) were elevated 8.6- and 6.2-fold, respectively, consistent with transactivation of RAR. Additionally, the RAR-target gene lecithin∶retinol acyltransferase (*Lrat*), which catalyzes the synthesis of retinyl esters from free retinol, was elevated 3.9-fold. Consistent with increased *Lrat* expression, levels of retinyl esters were 2.4-fold elevated in skin of SKO mice (**Figure 2B**). Skin has also been shown to possess physiologically significant acyl-CoA∶retinol acyltransferase (ARAT) activity due to acyl-CoA∶diacylglycerol acyltransferase 1 (DGAT1) and possibly other acyltransferases [47], [48]. Levels of *Dgat1* mRNA were decreased in the skin of SKO mice, consistent with a reduced synthesis of ARAT-derived retinyl esters (**Table 9**).

**Table 12:** Retinol metabolism.

AFFY ID	Gene Symbol	Gene Name	FC	PP of DE	q-value
1427747_a_at	*Lcn2*	lipocalin 2	**27.69**	1.00	0.043
1448754_at	*Rbp1*	retinol binding protein 1, cellular	**8.62**	1.00	0.019
1451191_at	*Crabp2*	cellular retinoic acid binding protein II	**6.20**	1.00	0.029
1416612_at	*Cyp1b1*	cytochrome P450, family 1, subfamily b, polypeptide 1	**4.97**	1.00	0.056
1444487_at	*Lrat*	lecithin-retinol acyltransferase (phosphatidylcholine-retinol-O-acyltransferase)	**3.87**	1.00	0.072
1422789_at	*Aldh1a2*	aldehyde dehydrogenase family 1, subfamily A2	**3.86**	1.00	0.116
1426968_a_at	*Rdh10*	retinol dehydrogenase 10 (all-trans)	**3.70**	1.00	0.053
1416613_at	*Cyp1b1*	cytochrome P450, family 1, subfamily b, polypeptide 1	**3.04**	1.00	0.053
1422723_at	*Stra6*	stimulated by retinoic acid gene 6	**2.79**	1.00	0.113
1417400_at	*Rai14*	retinoic acid induced 14	**2.58**	1.00	0.079
1448789_at	*Aldh1a3*	aldehyde dehydrogenase family 1, subfamily A3	**2.48**	0.99	0.176
1431010_a_at	*Rdh12*	retinol dehydrogenase 12	**2.38**	0.92	0.189
1417401_at	*Rai14*	retinoic acid induced 14	**2.24**	1.00	0.088
1424256_at	*Rdh12*	retinol dehydrogenase 12	**2.09**	1.00	0.059
1441030_at	*Rai14*	retinoic acid induced 14	**2.02**	1.00	0.111
1427395_a_at	*Aldh1a3*	aldehyde dehydrogenase family 1, subfamily A3	**1.82**	1.00	0.076
1422846_at	*Rbp2*	retinol binding protein 2, cellular	**1.62**	1.00	0.093
1417642_at	*Aldh1a3*	aldehyde dehydrogenase family 1, subfamily A3	**1.57**	0.97	0.115
1444777_at	*Rai14*	retinoic acid induced 14	**1.50**	1.00	0.053
1424715_at	*Retsat*	retinol saturase (all trans retinol 13,14 reductase)	**0.66**	0.65	0.154
1418808_at	*Rdh5*	retinol dehydrogenase 5	**0.65**	1.00	0.083
1427963_s_at	*Rdh9*	retinol dehydrogenase 9	**0.58**	1.00	0.062
1448326_a_at	*Crabp1*	cellular retinoic acid binding protein I	**0.58**	0.94	0.131
1450110_at	*Adh7*	alcohol dehydrogenase 7 (class IV), mu or sigma polypeptide	**0.57**	1.00	0.039
1449461_at	*Rbp7*	retinol binding protein 7, cellular	**0.56**	1.00	0.09
1452358_at	*Rai2*	retinoic acid induced 2	**0.52**	1.00	0.061
1441094_at	*Adh6b*	alcohol dehydrogenase 6B (class V)	**0.45**	1.00	0.055
1421702_at	*Rdh1*	retinol dehydrogenase 1 (all trans)	**0.43**	1.00	0.091
1426225_at	*Rbp4*	retinol binding protein 4, plasma	**0.42**	1.00	0.07
1416225_at	*Adh1*	alcohol dehydrogenase 1 (class I)	**0.27**	1.00	0.072
1422217_a_at	*Cyp1a1*	cytochrome P450, family 1, subfamily a, polypeptide 1	**0.19**	1.00	0.023
1415994_at	*Cyp2e1*	cytochrome P450, family 2, subfamily e, polypeptide 1	**0.16**	1.00	0.094
1416468_at	*Aldh1a1*	aldehyde dehydrogenase family 1, subfamily A1	**0.14**	1.00	0.089
1429608_at	*Adh6a*	alcohol dehydrogenase 6A (class V)	**0.09**	1.00	0.019

See Table 3 for legend.

Elevation of RAR target gene expression in skin is consistent with increased levels of retinoic acid in the skin. Indeed, the levels of all-*trans* retinol and all-*trans* retinoic acid were elevated in SKO skin 2.3-fold and 1.9-fold, respectively (**Figure 2A**). Plasma levels of all-*trans* retinol (**Figure 2C**), as well as retinyl ester stores in the liver (**Figure 2D**) and epididymal white adipose (data not shown) were not significantly different between Lox and SKO mice.

Retinol is oxidized to retinaldehyde by cytosolic alcohol dehydrogenases (ADH) of the medium-chain dehydrogenase/reductase family and microsomal retinol dehydrogenases (RDH) of the short-chain dehydrogenase/reductase family [49]. We observed decreased expression of *Adh1* (class I), *Adh7* (Class 4), *Adh6a* and *Adh6b* (class V), as well as decreased expression of *Rdh1*, *Rdh5* and *Rdh9*, but increased expression of *Rdh10* and *Rdh12* (**Table 12**). Retinaldehyde is converted to retinoic acid via the action of retinaldehyde dehydrogenases encoded by *Aldh1a1–Aldh1a3*. The expression of the retinaldehyde dehydrogenase genes *Aldh1a2* and *Aldh1a3* were elevated, whereas the expression of *Aldh1a1* was decreased (**Table 12**). We also observed 2.8-fold increase expression of *Stra6*, which encodes the membrane receptor for the circulating retinol binding protein [50]. A decreased capacity for retinoic acid catabolism may also contribute to the elevated levels of skin retinoic acid. We observed a robust decrease in the expression of *Cyp1a1* and *Cyp2e1*, but increased expression of *Cyp1b1*, all of which are expressed in murine skin and encode enzymes with reported retinoic acid 4-hydroxylase activity (**Table 12**) [51], [52].

To help determine whether the disturbed retinol metabolism is responsible for the skin phenotypes in *Scd1* SKO mice, we subjected Lox and SKO mice to a retinoid-deficient (RD) diet intervention. In both Lox and SKO mice, this RD diet intervention dramatically reduced hepatic retinol and retinyl ester stores to less than 0.25% of the hepatic stores observed in mice fed the standard diet (**Figure 2D**), but in comparison only modestly reduced plasma retinol levels in both Lox and SKO mice (**Figure 2C**). However, skin all-*trans* retinoic acid levels remained 1.5-fold elevated in SKO mice compared to Lox mice (p<0.05; data not shown). Gross and histological examination of skin indicated that the RD diet elicited no remarkable improvement in the phenotype observed in standard diet-fed SKO mice (data not shown). This highlights that SCD1 is critical for skin retinoid homeostasis even under conditions of reduced retinol availability.

### Retinoic acid transactivation of PPARδ

Retinoic acid can bind to both CRABP2 and FABP5, which then delivers the ligand to transactivate RAR and PPARδ, respectively [53], [54]. The relative ratio of FABP5 to CRABP2 is an important determinant of the relative transactivation of RAR and PPARδ [53], [54]. In addition to the aforementioned increased expression of *Ppard* and *Crabp2* in SKO mice, we observed elevated *Fabp5* (**Table 9**), which combined with increased retinoic acid availability may lead to increased PPARδ transactivation. PPARδhas been shown to contribute to the expression of *Lrat* and *Rbp1* in activated hepatic stellate cells and may also influence the expression of retinoid metabolism genes in the skin [55]. Interestingly, transgenic mice that overexpress PPARδ develop a psoriasis-like inflammatory skin disease upon ligand activation featuring hyperproliferation of keratinocytes, dendritic cell accumulation, and endothelial cell activation [41]. Consistent with this model, we observed a 10-fold elevation in SKO mice of the direct PPARδtarget heparin-binding EGF-like growth factor (*Hbegf*; **Table 7**), which is elevated in psoriasis and induces epidermal hyperplasia [56]. The EGF-family ligand TGF-α (*Tgfa*) and the kinase *Ptk6*, which are also increased in both psoriasis and PPARδ transgenic mice, were elevated in SKO mice as well (**Table 7**) [41]. Thus, the elevated retinoic acid levels in the skin of SKO mice may be transactivating both RAR and PPARδ.

### Inhibition of SCD in SEB-1 sebocytes elevates retinoic acid-induced genes including *LCN2*

**Figure 3:**
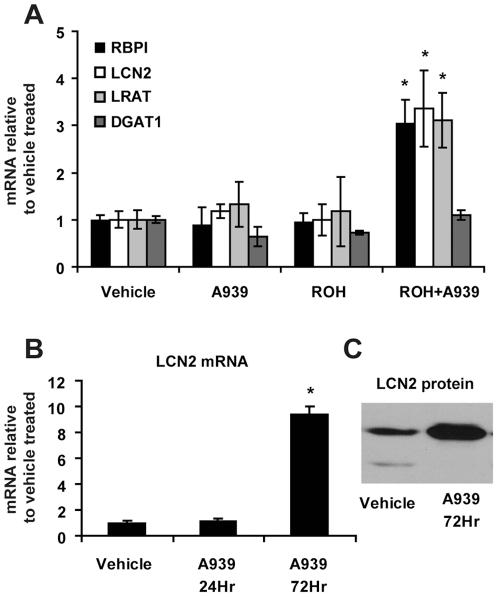
Loss of SCD1 activity in SEB-1 sebocytes increases retinoic acid target gene induction. A) SEB-1 human sebocytes were treated with vehicle alone, the SCD inhibitor A939572 (A939; 1 µM), free retinol (ROH; 1 µM), or both A939 and ROH for 18 hours. Total RNA was collected for assessment of expression of *RBP1*, *LCN2*, *LRAT* and *DGAT1*. Data are expressed relative to vehicle treated cells. B) SEB-1 cells were grown for the indicated times in the presence or absence of A939572 (A939; 1 µM) and total RNA was collected for assessment of *LCN2* induction. C) Cellular protein expression of LCN2 increased after 72 hour treatment with A939 as well. Data represent mean ± SEM for n = 3 per group. * = *p*<0.05 vs. vehicle control.

Interestingly, the skin of SKO mice displayed a 27.7-fold elevation in the expression of *Lcn2* (**Table 12**), which has previously been shown to be a retinoic acid-induced gene linked to sebocyte apoptosis in human SEB-1 cells [57]. We used the immortalized human sebocyte line SEB-1 as a model to assess the role of SCD in regulating retinol metabolism in vitro. SEB-1 sebocytes have measurable SCD activity that can be inhibited by the small molecule A939572 (**Figure S1**). SEB-1 cells treated with 1 µM A939572 and 1 µM retinol for 24 h displayed a 3-fold increase in the retinoic acid-induced target genes *RBP1*, *LRAT* and *LCN2* (**Figure 3A**). These genes were not elevated in cells treated for 24 h with 1 µM retinol alone. Although treatment with A939572 alone for 24 h did not elevate *LCN2*, prolonged treatment for 72 h caused a 9-fold upregulation of *LCN2* mRNA and protein (**Figure 3B and 3C**). The difference between 24 and 72 hours may be due to cellular retinol accumulation following media replacement at 48 hours. At all retinol concentrations tested (0.01 to 10 µM), LCN2 protein secreted into the media was higher in cells treated with A939572 (**Figure S1**). As expected, the expression of *DGAT1* was unaffected by retinol and/or A939572 treatment. These data support a direct role of sebocyte SCD activity in controlling retinol metabolism to prevent an increase in cellular retinoic acid and RAR-regulated target genes.

### Changes in retinoic acid and inflammatory genes occur in the first hair cycle

**Figure 4:**
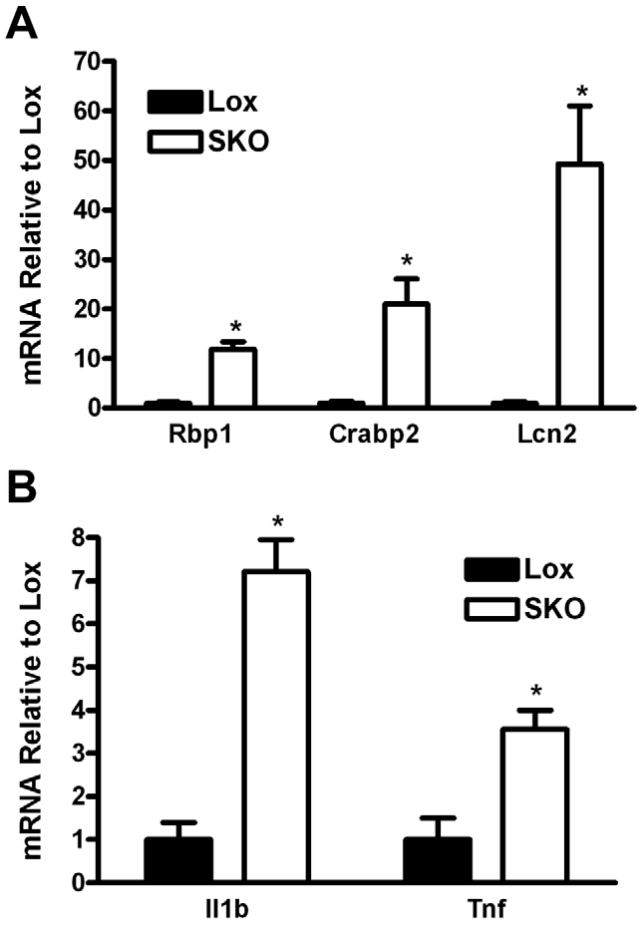
Elevated retinoic acid metabolism and inflammatory genes in 23 day old SKO skin. RNA was isolated from skin of 23 day old postnatal Lox and SKO mice and subjected to real-time PCR analysis and normalized to levels of *18S* as described in *Methods*. The expression of A) retinoic-acid regulated genes (*Lcn2*, *Rbp1*, *Crabp2*) and B) inflammation genes (*Il1b*, *Tnf*) were significantly elevated in SKO mice at 23 days. All values are expressed as fold difference relative to Lox mice. Data represent mean ± SEM for n = 4–6 per group. * = *p*<0.05.

Hair follicle cycling begins at around 17 days postnatal in mice, characterized by the occurrence of the first catagen and telogen stages, and followed by the first anagen growth stage at around 4 weeks after birth [58], [59]. *Scd1* expression has previously been shown to be low at birth, increase dramatically at around 8 days postnatal during the completion of hair follicle morphogenesis and then cycle between high and low expression during the anagen and catagen/telogen phases of the hair cycle, respectively [6]. We analyzed the expression pattern of retinoic acid-regulated genes (*Rbp1*, *Crabp2* and *Lcn2*) and inflammatory genes (*Il1b* and *Tnf*) in 23 day postnatal skin between Lox and SKO. Consistent with our microarray observations in older mice, these retinoic acid-regulated and inflammatory genes were significantly elevated in SKO mice (**Figure 4**). This highlights that disturbed retinoic acid metabolism and inflammation in SKO mice precedes adulthood and may be the primary insults causing local and systemic changes in these mice.

## Discussion

The primary goal of this study was to determine the mechanism by which reduced sebaceous gland MUFA production elicits a profound hypermetabolic whole-body phenotype. Mice with a targeted deletion of *Scd1* have a dysfunctional epidermal lipid barrier that results in increased transepidermal water loss (TEWL) and has been suggested to be the source of the hypermetabolism [8], [13]. Interestingly, artificial occlusion of the skin of *Scd1*-deficient mice with topical application of petroleum jelly largely reversed the cold intolerance, high TEWL and increased O_2_ consumption and CO_2_ production [13]. We investigated whether maintaining SKO mice at thermoneutral temperature would correct the hypermetabolic phenotype and render the SKO mice susceptible to diet-induced obesity. However, SKO mice remained lean and hyperphagic, similar to those maintained at ambient temperature [7]. Although the elevated temperature in the thermoneutral environment likely negates heat loss due to convection, conduction and radiation, the humidity in the thermoneutral environment (30–40%) may have still permitted heat loss due to evaporation that contributed to the obesity resistance.

Interestingly, *Scd1^ab-2J^*, but not *Scd1^ab-J^* mice, with naturally occurring mutations in *Scd1* also have increased TEWL similar to mice with a targeted deletion of *Scd1* [8], [13]. Studies that directly compared the *Scd1^ab-2J^* and *Scd1^ab-J^* mice reported that although both strains have increased dermal inflammation and reduced stratum corneum hydration, these phenotypes are more severe in *Scd1^ab-2J^* compared to the *Scd1^ab-J^* mice [5], [8]. These results suggest inflammation and/or stratum corneum hydration may be involved in the TEWL phenotype, but that other genetic modifiers may influence the onset and severity of these phenotypes due to *Scd1* deficiency. Treatment of *Scd1^ab^* mice with the immunosuppressant drug cyclosporine A reduces inflammation, epidermal thickness and hyperkeratosis, and restored some hair growth [60]. We observed a remarkable enrichment of genes involved in the inflammatory process, supporting the involvement of inflammation in the progression of the skin phenotype. Since TEWL is known to increase under conditions of skin irritation and dermatitis [61], [62], it is possible that the primary mechanism for the hypermetabolism is due to a pro-inflammatory stimulus in the skin.

The decreased stratum corneum hydration in *Scd1*-deficient mice has been suggested to be due to reduced glycerol content that results from diminished sebaceous gland-derived triglyceride hydrolysis [5]. In support of this model, we observed robust repression of genes encoding transcription factors and enzymes involved in fatty acid and triglyceride synthesis, triglyceride hydrolysis and sebocyte differentiation. Although there may be less sebaceous gland-derived glycerol due to *Scd1* deficiency, we observed a marked increase in *Aqp3* and *Aqp9*, two aquaglyceroporins essential for normal glycerol transport and metabolism [63], [64] (**Table S2**). This may serve as an adaptation to low glycerol availability to help maintain normal glycerol status. It has also been suggested that a deficiency of epidermal ceramides containing very long-chain fatty acids, due to decreased elongase expression, contributed to the epidermal barrier disruption [13]. Consistent with these results, we also found a dramatic repression of several elongase genes. Combined with an overall reduced capacity for fatty acid synthesis, the reduced elongase expression would be predicted to severely compromise very long chain fatty acid synthesis.

We unexpectedly found an elevation in skin retinol and retinoic acid levels as well as a marked elevation in retinoic-acid responsive genes due to skin *Scd1* deficiency. This is reminiscent of *Dgat1*-deficient mice, which have been shown to have a reduced capacity for skin retinol esterification leading to elevated levels of skin retinol and retinoic acid, and resulting in cyclical hair loss [48]. Although LRAT is the major retinyl ester synthesizing enzyme in most cells, in vivo studies by O'Byrne *et al.* in *Lrat*-deficient mice support the existence of a physiologically significant ARAT activity for retinyl ester formation [65]. Indeed, Wongsiriroj *et al.* first confirmed the physiological relevance of an ARAT activity for DGAT1 using mice deficient in both *Lrat* and *Dgat1* [66]. Kurlandsky *et al.* proposed that LRAT acts primarily in the basal keratinocytes of the skin, whereas retinyl ester synthesis in the differentiating suprabasal keratinocytes is predominantly derived from an ARAT activity [47]. Therefore, certain cells in the skin, such as the suprabasal keratinocytes and possibly sebocytes, may have a greater dependency on ARAT activity for retinyl ester formation. We speculate that MUFA, which are important substrates for DGAT1-mediated triglyceride formation, are also an important substrate for retinyl ester formation in cells that have a greater reliance on ARAT activity for retinyl ester synthesis.

Since our studies are performed in whole-skin, the retinyl esters, retinol and retinoic acid might all be compartmentalized in different places. The accumulated retinol in the skin of SKO mice may be localized to a compartment that is inaccessible to LRAT, and subsequently converted to retinoic acid. Furthermore, the accumulated retinoic acid could possibly diffuse out and upregulate LRAT-mediated retinyl ester formation in different cells. Alternatively, retinyl ester hydrolase(s) may become active, resulting in a futile cycle where retinyl esters are made and then hydrolyzed. The persistent elevation in skin retinoic acid levels could also be due to decreased retinoic acid catabolism. We observed robust suppression of *Cyp2e1* and *Cyp1a1*, which encode two cytochrome P450 enzymes that can catalyze retinoic acid 4-hydroxylase activity in murine skin [51], [52].

Despite reducing liver retinol stores by more than 400-fold with a RD diet intervention in both Lox and SKO mice, we were unable to normalize skin retinoic acid levels and normal hair growth in SKO mice. In contrast, studies by Shih *et al.* in *Dgat1*-deficient mice were able to normalize skin retinoic acid levels and hair growth with a similar intervention [48]. This may indicate that lack of *Scd1* causes a more pronounced alteration in retinoic acid metabolism. Consistent with this notion, the skin phenotype elicited by *Scd1* deficiency is more severe than that observed due to *Dgat1* deficiency [48]. Alternatively, non-retinoid pathways may also be contributing to the skin phenotype. MUFAs are also important for cellular cholesterol ester synthesis [11] and SKO mice have elevated levels of skin free cholesterol [18]. Thus, a decrease in MUFA availability may affect both cholesterol and retinol homeostasis, in addition to triglyceride synthesis.

The therapeutic and pathological effects of retinoids on the skin have been known for many years. In vivo, both topical and orally administered retinoids stimulate keratinocyte proliferation and differentiation, resulting in increased number of epidermal cell layers and epidermal thickness, widening of spaces between keratinocytes and changes in the thickness and organization of the stratum corneum [67], [68], [69]. Furthermore, retinoid treatment causes a dose-dependent increase in TEWL, potentially due to loosening and fragility of the stratum corneum [68], [70]. The high responsiveness of various skin cells, such as epidermal keratinocytes, follicular keratinocytes, and sebocytes, to retinoid treatment is explained by their prominent expression of enzymes, binding proteins, and nuclear receptors involved in retinoic acid synthesis and signaling [71].

One of the most well-studied retinoid drugs is isotretinoin (13-*cis* retinoic acid), which likely acts as a prodrug that becomes selectively activated in the sebocyte possibly after isomerization to tretinoin (all-*trans* retinoic acid) [72]. Isotretinoin treatment causes apoptosis and reduced size of the sebaceous gland, and the sebocytes appear undifferentiatied and have decreased lipid accumulation [2], [73]. We propose that the elevated levels of all-*trans* retinoic acid in the skin of SKO mice leads to sebaceous gland dysfunction. As previously suggested by Sundberg et al., the sebaceous gland dynsfunction due to *Scd1*-deficiency impairs the degradation of the inner root sheath causing restraint and destruction of the hair follicle, inducing an inflammatory reaction, epidermal hyperplasia and scarring alopecia [8].

Skin biopsies taken from patients treated with isotretinoin also showed high expression of *LCN2* (lipocalin-2) [57], [73]. Additionally, isotretinoin treatment of SEB-1 human sebocyte cultures causes cell cycle arrest and apoptosis concomitant with increased expression of *LCN2* [72], [74]. SEB-1 cells were rescued from isotretinoin-induced apoptosis upon siRNA knockdown of *LCN2* [57]. The *LCN2* promoter has binding sites for both RAR and retinoid-X-receptor (RXR), suggesting that the toxic effect of retinoic acid on sebocytes is occurring via a lipocalin-2-mediated mechanism [57]. Since we observed a robust 27.7-fold elevation of *Lcn2* in the skin of SKO mice, it is possible that the primary disturbance in the skin of SKO mice is retinoic acid-induced *Lcn2* leading to sebocyte dysfunction and sebaceous gland hypoplasia. However, *Lcn2* is also increased in lesional compared to non-lesional skin samples from psoriasis patients, as well as increased in the skin of a murine model of PPARδ hyperactivation with a psoriasis-like condition [41]. Therefore, the increased *Lcn2* in SKO mice may be influenced not only by retinoic acid, but also by the secondary effects of inflammation [75]. Alternatively, decreased expression of fatty acid metabolizing enzymes and key transcription factors involved in lipid synthesis in SKO mice may also contribute to sebaceous gland hypoplasia. Future studies are required to ascertain which of these potential mechanisms is responsible for the skin phenotype of the SKO mice.

In summary, we have shown that SCD1 is essential for normal retinoid homeostasis in the skin. Lack of skin SCD1 causes an elevation in skin levels of retinol and retinoic acid, which in turn activates the transcription of retinoic acid-induced genes. We speculate that one of these elevated retinoic acid-induced genes, *Lcn2* (lipocalin-2) results in sebocyte dysfunction and sebaceous gland hypoplasia in the SKO mice. Additionally, lack of SCD1 causes decreased fatty acid synthesis gene expression in the skin concomitant with elevated cholesterol synthesis. This pattern suggests a state of disturbed lipid metabolism that may also contribute to the skin phenotype. The disturbed retinol metabolism and inflammatory gene expression is also evident at 23 days postnatal, highlighting that *Scd1* is essential for early events in sebocyte development and normal hair cycling.

## Methods

### Animals and diets

*Scd1^flox/flox^* (Lox) and *Scd1^flox/flox^;Krt14-Cre/+* (skin-specific *Scd1* knockout; SKO) mice on a C57BL/6J background were generated as previously described [7]. All mice used in this study were male unless otherwise stated. Mice were housed in a controlled environment (21°C; 30–40% relative humidity) with 12 h light and dark cycles and fed a standard diet (PMI 5008 Formulab; Purina Mills Nutrition International, Richmond, Indiana) unless otherwise stated. Animal experiments were approved by the Animal Care Research Committee of the University of Wisconsin-Madison under protocol A00625. For diet-induced obesity experiments under thermoneutral conditions, mice were transferred at 6 weeks of age to a controlled environment (32.5–33.5°C; 30–40% relative humidity), allowed to acclimate for 2 weeks and then fed a high-fat diet (Harlan TD.06414; 60% kcal fat) for 7 weeks. Retinol deficiency was induced by feeding pregnant dams a retinoid-deficient (RD) diet (Harlan TD.09238; 10% kcal fat; <0.04 IU retinol/g) throughout gestation and suckling. The third consecutive litter from dams maintained continuously on the RD diet were weaned and maintained on the RD diet until approximately 10 weeks of age.

### Collection of gene expression data

Dorsal skin of 8 to 9 week-old non-fasted male mice fed a standard diet was shaved with electric clippers prior to sacrifice. Mice were sacrificed by isoflurane overdose in the early light cycle and dorsal skin, liver, adipose tissue and plasma were immediately frozen in liquid nitrogen. Total RNA was extracted from whole skin using TRI reagent (Molecular Research), and treated with TURBO DNase (Ambion) before being subjected to microarray studies. Affymetrix Mouse 430 2.0 microarray chips were used to monitor the expression level of 45,101 probe sets representing over 39,000 transcripts and variants from over 34,000 mouse genes (Affymetrix). All microarray data are MIAME compliant and are deposited as Gene Expression Omnibus accession GSE24243, which may be accessed at http://www.ncbi.nlm.nih.gov/geo. Gene abbreviations are defined in accordance with the Human Gene Nomenclature Committee [76]. RNA isolated from 23 day old skin samples or SEB-1 cell cultures was reverse transcribed using Mutliscribe reverse transcriptase (Applied Biosystems) and subjected to real-time PCR on the ABI Prism 7500 Fast instrument using Power SYBR Green master mix (Applied Biosystems) and *18S* as a normalization gene. The primer sequences are available upon request.

### Pre-Processing and Statistical Analysis of Gene Expression Data

Expression measurements were pre-processed to provide background correction, normalization and log base 2 transformation using RMA (Robust Multi-array Average) [77]. We used two analytical approaches to generate lists of differentially expressed probe sets, loosely referred to as genes. First, we calculated an un-moderated t-statistic for every probe set by applying Welch's t-test. The resulting p-values were used to calculate q-values, which account for multiple tests and provide thresholds so that lists can be generated for a target false discovery rate (FDR) [78]. Using this method, we targeted a FDR of 5% and probe sets with q-values below 0.05 are considered to be of interest.

We also applied EBarrays [79], [80], which along with RMA is implemented in R, a publicly available statistical analysis environment [81] and available at Bioconductor [82]. EBarrays is an empirical Bayes approach which models the probability distribution of a set of expression measurements [79], [80]. It accounts generally for differences among probe sets in their true underlying expression levels, measurement fluctuations and distinct expression patterns for a given probe set among conditions [79]. An expression pattern is an arrangement of the true underlying intensities (μ) in each condition. The number of patterns possible depends on the number of conditions from which the expression measurements were obtained. For example, when measurements are taken from two conditions, two patterns of expression are possible: equivalent expression (EE; μ_1_ = μ_2_) and differential expression (DE; μ_1_≠μ_2_). Since we do not know a priori which probe sets are in which patterns, the marginal distribution of the data is a mixture over the possible patterns with model parameters determined by the full set of array data. In this way, the approach utilizes information across a set of arrays to optimize model fit and is thus more efficient than a number of methods that make inferences one probe set at a time [79]. The approach also naturally controls for both type I and type II errors [79]. The fitted model parameters provide information on the number of probe sets expected in each expression pattern. Furthermore, the fitted model is used to assign posterior probability distributions to every probe set. Each probe set specific distribution gives the posterior probability of that probe set's individual expression pattern. The posterior expected FDR is controlled by thresholding the posterior probabilities. Two approaches were used as discussed in [83]. The most conservative approach to control the FDR at 5%, for example, is to only consider probe sets with specific posterior probability of EE less than 0.05 (hard threshold). We also report results from the less conservative approach that determines the exact threshold required (soft threshold) so that the average posterior probability of EE for all probe sets on a list is less than 0.05.

Tests for enrichment of common function among sets of differentially-expressed probe sets were carried out using data from the Gene Ontology (GO) annotations and the Kyoto Encyclopedia of Genes and Genomes (KEGG). The R package allez was be used to perform tests of enrichment for each GO category and KEGG pathway [84]. In general, the interpretation of p-values resulting from hypergeometric-based enrichment tests is not straightforward due to the many dependent hypotheses tested. Furthermore, the enrichment test tends to result in small p-values when groups with few probe sets are considered. The statistical methods underlying allez adjust for these factors, allowing increased power and sensitivity for identifying sets that are biologically meaningful. We considered sets with between 5 and 500 probe sets and identified a set as enriched if its Z-score exceeded 5, as recommended in [84].

### High performance liquid chromatography (HPLC) analysis of retinoids

Reverse phase HPLC analysis of retinol and retinyl esters in tissues was performed as described elsewhere [85], [86]. Briefly, all samples were flash frozen in liquid N_2_ immediately after collection. Liver, skin and adipose tissues were first homogenized in 10 volumes of PBS (10 mM sodium phosphate, pH 7.2, 150 mM sodium chloride) using a Polytron homogenizer (Brinkmann Instruments, Westbury, NY) set at half-maximal speed for 10 s. 200 µl of the homogenate or 100–200 µl of plasma was treated with an equal volume of absolute ethanol containing a known amount of retinyl acetate as an internal standard, and the retinoids present in the homogenates were extracted into hexane. The extracted retinoids were separated on a 4.6×250-mm Ultrasphere C_18_ column (Beckman, Fullerton, CA) preceded by a C_18_ guard column (Supelco, Bellefonte, PA), using 70% acetonitrile-15% methanol-15% methylene chloride as the running solvent, flowing at 1.8 ml/min. Retinol and retinyl esters (routinely, retinyl palmitate, oleate, linoleate, stearate) were identified using a Waters 2996 photodiode array detector to compare retention times and spectral data of experimental compounds with those of authentic standards. Concentrations of retinol and retinyl esters in these samples were quantitated by comparing integrated peak areas of the unknowns against those of known amounts of purified standards. An internal standard, retinyl acetate, added immediately after homogenization of the samples, was used to correct for losses during extraction.

### Determination of tissue levels of all-*trans*-retinoic acid

Tissue levels of all-*trans*-retinoic acid were determined by ultra performance liquid chromatography tandem mass spectrometry (UPLC/MS/MS). For this purpose, we employed LC/MS grade acetonitrile, hexane, and water purchased from Fisher Scientific (Pittsburgh, PA). All-*trans*-retinoic acid was purchased from Sigma-Aldrich. Penta-deuterated all-*trans*-retinoic acid (atRA-d5) was employed as an internal standard and was purchased from Toronto Research Chemicals (North York, Ontario). Retinoid concentrations of standards were verified spectrophotometrically using published ε values [87]. Tissue homogenates were extracted using the two-step acid-base extraction described by Kane *et al.* [88]. Briefly, 0.5 ml of 0.025 M KOH in ethanol was added to 250 µl tissue homogenate prepared from 50 mg of wet tissue. Five ng of atRA-d5 dissolved in absolute ethanol was added to each tissue homogenate as internal standard. Retinoids present in this mixture were extracted into 5 mL of hexane (LC/MS grade, Fisher). Following centrifugation to separate phases, the upper organic phase containing nonpolar retinoids (retinol and retinyl esters) was removed. Thirty µl of 4 M HCl was then added to the aqueous phase and polar retinoids like all-*trans*-retinoic acid were extracted into 5 mL hexane. The retinoid containing organic phase was removed and dried under nitrogen. The dried extract was resuspended in 70 µl of acetonitrile (Fisher) and transferred to an amber LC/MS vial (Waters). Only glass containers, pipettes, and calibrated syringes were used to handle retinoic acid.

UPLC/MS/MS analyses were carried out on an Waters Xevo TQ MS ACQUITY UPLC system (Waters, MA). The UPLC/MS/MS system was controlled by MassLynx Software 4. 1. Samples were maintained at 4°C in the autosampler and 5 µl was loaded onto a Waters ACQUITY UPLC HHS C18 column (2.1 mm×100 mm, 1.8 µm particles) preceded with a 2.1 mm×5 mm guard column containing the same packing material (Waters). Throughout chromatography, the column was maintained at 40°C. The flow rate was 300 µl/min employing a binary gradient the following mobile phase gradient which was initiated with 32% phase A consisting of H_2_O (LC/MS grade, Fisher), containing 0.1% formic acid, and 68% mobile phase B consisting of acetonitrile, containing 0.1% formic acid. Initial solvent conditions were maintained for 6.3 minutes, at which time, the percentage of solvent B was increased linearly to 85% by 6.4 min. This solvent mixture was maintained until 9.5 min when the percentage of solvent B was increased to 100% to wash the column. The wash was allowed to continue for 2 min and then flow was returned to the initial binary gradient consisting of 68% solvent B. All-*trans*-retinoic acid eluted between 8.2 and 8.4 min. Positive electrospray ionization mass spectrometry was performed using the following parameters: capillary voltage 3.8 kV, source temperature 150°C, dissolving temperature 500°C, dissolving gas flow 800 L/hr, collision gas flow 0.15 ml/min. The optimized cone voltage was 16 V and the collision energy for the multiple reactions monitoring mode (MRM) was 18 eV. All-*trans*-retinoic acid was detected and quantified using MRM employing the following transitions: all-*trans*-retinoic acid, 301.16→123.00 m/z; and atRA-d5, 306.15→127.03 m/z.

### SEB-1 Cell Culture

Cells from the SEB-1 human sebaceous gland primary cell line (obtained from Dr. Diane M. Thiboutot, Penn State Milton S. Hershey Medical Center) were maintained in DMEM with 10% FBS and 1% penicillin/streptomycin at 37°C and 5% CO_2_. Cells were grown in standard culture conditions until confluence, after which time the medium was replaced with serum free medium to eliminate exogenous free fatty acids, notably FBS mediated 18∶1 delivery. Cells were treated either with 1 µM or with the indicated concentrations of free retinol in serum free DMEM for 18 hours, after which time total RNA was collected and used for assessment of retinoic acid-mediated gene induction. Free retinol was dissolved in EtOH in a 10 mM stock under yellow light with all subsequent dilutions and treatment of cells protected from direct white light. SCD was inhibited using the small molecule inhibitor (4-(2-chlorophenoxy)-N-(3-(methylcarbamoyl)-phenyl)piperidine-1-carboxamide) (A939572) from Biofine International Inc. (Vancouver, BC, Canada). Working concentrations (1 µM) were diluted into serum free DMEM prior to assay. Vehicle (control) treated cells consisted of diluent (DMSO) in serum free DMEM. Lipocalin-2 protein was detected in cell lysates and media using a rabbit polyclonal antibody against lipocalin-2 (ab63929) from Abcam.

### Quantification and inhibition of SCD activity in SEB-1 cells

SEB-1 cells were treated with increasing concentrations of stearate ranging from 0–100 µM stearic acid containing 0.5 µCi ^14^C stearic acid conjugated to BSA in DMEM containing 1% FBS for 4 hours. Cells were then collected into 400 µl NaOH and after addition of 400 µl butylated hydroxytoluene (1.25 mg/ml in EtOH), cellular lipids were saponified at 85°C for 1 hour. The mixture was acidified by addition of 540 µl formic acid, and lipids extracted with 700 µl hexane. Free fatty acids were resolved on a 10% AgNO_3_-impregnated silica gel TLC plate using chloroform∶methanol∶acetic acid∶H_2_O (90∶8∶1∶0.8). Radioactive spots were read using a Packard Instant Imager with the upper band corresponding to ^14^C stearate and the lower band ^14^C oleate. Quantification of SCD-1 activity was determined based on the intensity of ^14^C oleate (lower band) formed relative to the ^14^C stearate (upper band). Cells were treated with or without the small molecule SCD inhibitor A939572 (1 µM; Biofine).

#### Other statistical analyses

Values are reported as mean ± SEM and were compared by *t* test or analysis of variance followed by Bonferroni or Tukey's Post-hoc test.

## Supporting Information

Figure S1 **Characterization of SCD1 activity and lipocalin-2 secretion in human SEB-1 sebocytes.** A) The relative activity of endogenous SCD1 was measured in SEB-1 sebocytes as described in *Methods*. SEB-1 sebocytes display measurable conversion of ^14^C-stearate into ^14^C-oleate that is able to be inhibited by the small molecule SCD1 inhibitor A939572. B) Increasing concentrations of free retinol were used to measure the appearance of LCN2 secreted into the media from SEB-1 cells. At low concentrations, cells with and without SCD1 activity demonstrated a modest increase in LCN2 secretion. Higher retinol levels (≥1 µM) did not increase LCN2 secretion in cells with SCD1 activity possibly due to feedback inhibition, whereas SCD1 inhibited cells continued to increase LCN2 secretion when treated with up to 10 µM retinol.
(PDF)

Table S1 **Inflammation, wound healing and defense response.** Changes in gene expression are reported as fold-change (FC) relative to Lox mice. Significant differences between Lox and SKO were determined as described in *Methods*, and for both Welch's t-test and EBarrays the false discovery rate was set at 5%. All probe sets listed have a posterior probability of differential expression (PP of DE) >0.639 (soft threshold) based upon analysis by EBarrays. Additionally, Welch's t-test was used to calculate q-values and those probe sets with q-values <0.05 were considered significant.
(PDF)

Table S2 **Miscellaneous categories.** Changes in gene expression are reported as fold-change (FC) relative to Lox mice. Significant differences between Lox and SKO were determined as described in *Methods*, and for both Welch's t-test and EBarrays the false discovery rate was set at 5%. All probe sets listed have a posterior probability of differential expression (PP of DE) >0.639 (soft threshold) based upon analysis by EBarrays. Additionally, Welch's t-test was used to calculate q-values and those probe sets with q-values <0.05 were considered significant.
(PDF)

Table S3 **Proteases and peptidases.** Changes in gene expression are reported as fold-change (FC) relative to Lox mice. Significant differences between Lox and SKO were determined as described in *Methods*, and for both Welch's t-test and EBarrays the false discovery rate was set at 5%. All probe sets listed have a posterior probability of differential expression (PP of DE) >0.639 (soft threshold) based upon analysis by EBarrays. Additionally, Welch's t-test was used to calculate q-values and those probe sets with q-values <0.05 were considered significant.
(PDF)

Table S4 **Collagens, keratins, gap junctions and tight junctions.** Changes in gene expression are reported as fold-change (FC) relative to Lox mice. Significant differences between Lox and SKO were determined as described in *Methods*, and for both Welch's t-test and EBarrays the false discovery rate was set at 5%. All probe sets listed have a posterior probability of differential expression (PP of DE) >0.639 (soft threshold) based upon analysis by EBarrays. Additionally, Welch's t-test was used to calculate q-values and those probe sets with q-values <0.05 were considered significant.
(PDF)
