# Supplementary figures and images for: Metabolic Changes in Skin Caused by Scd1 Deficiency: A Focus on Retinol Metabolism
Source: PLoS One. 2011 May 9;6(5):e19734. doi: 10.1371/journal.pone.0019734 (PMC3090422; doi:10.1371/journal.pone.0019734)

# Supplementary Figure 1

**A**

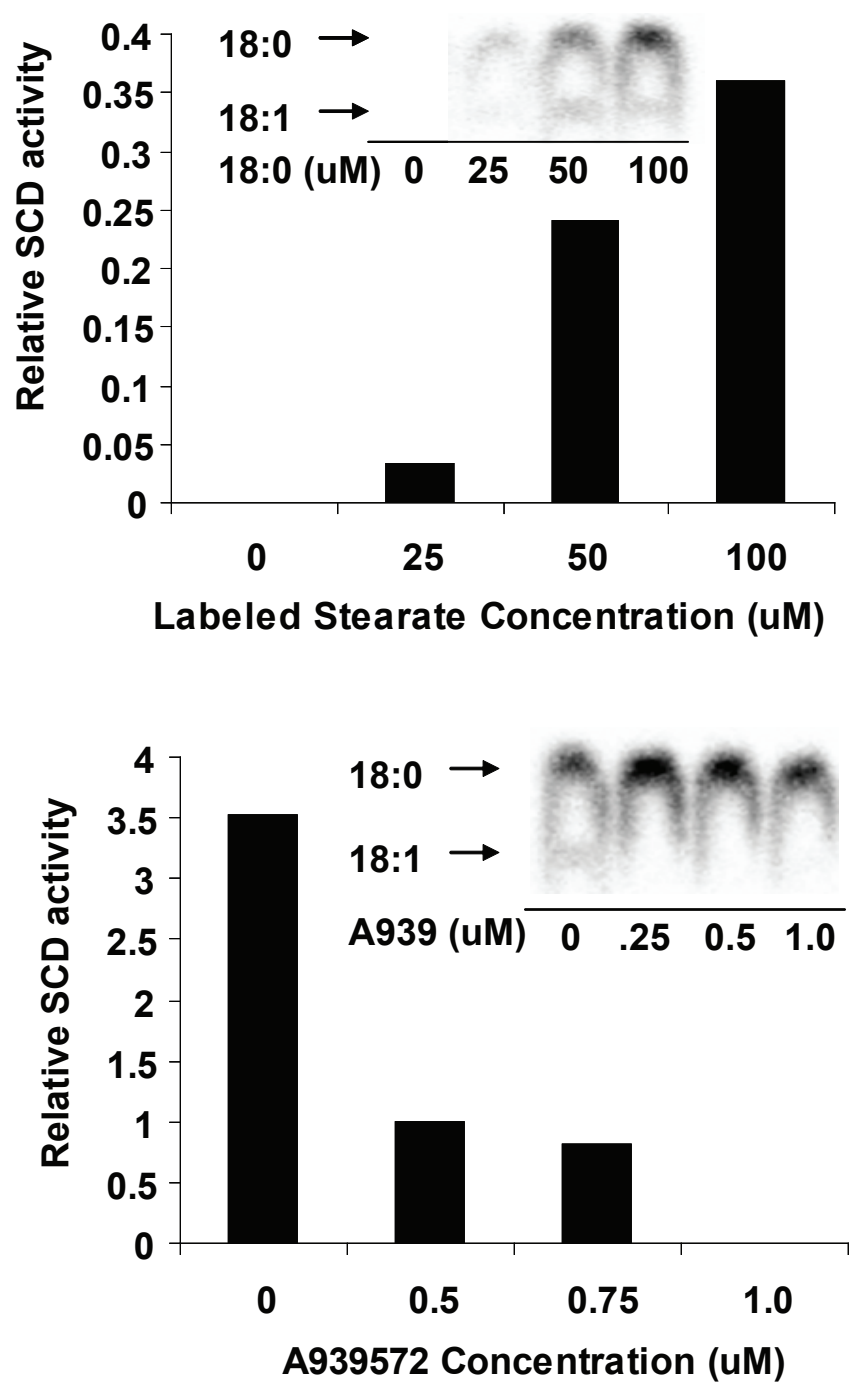

**B**

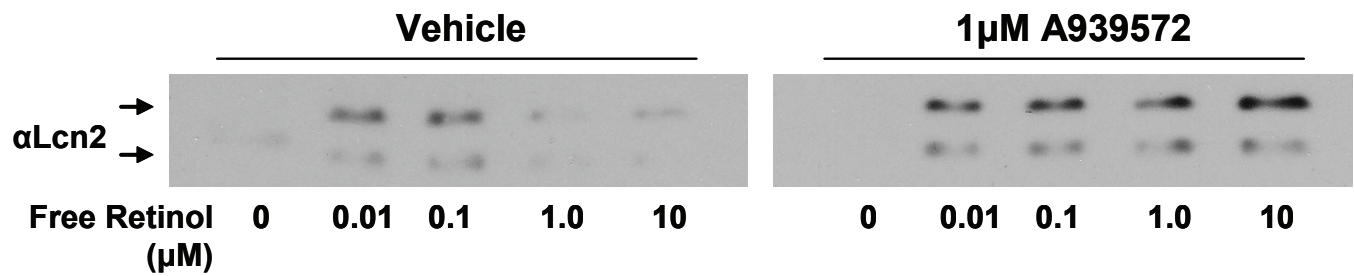

Supplement: Figure S1 — Characterization of SCD1 activity and lipocalin-2 secretion in human SEB-1 sebocytes. A) The relative activity of endogenous SCD1 was measured in SEB-1 sebocytes as described in Methods . SEB-1 sebocytes display measurable conversion of 14C-stearate into 14C-oleate that is able to be inhibited by the small molecule SCD1 inhibitor A939572. B) Increasing concentrations of free retinol were used to measure the appearance of LCN2 secreted into the media from SEB-1 cells. At low concentrations, cells with and without SCD1 activity demonstrated a modest increase in LCN2 secretion. Higher retinol levels (≥1 µM) did not increase LCN2 secretion in cells with SCD1 activity possibly due to feedback inhibition, whereas SCD1 inhibited cells continued to increase LCN2 secretion when treated with up to 10 µM retinol. (PDF) [file pone.0019734.s001.pdf]
