# Supplementary material for: Metabolic Changes in Skin Caused by Scd1 Deficiency: A Focus on Retinol Metabolism
Source: PLoS One. 2011 May 9;6(5):e19734. doi: 10.1371/journal.pone.0019734 (PMC3090422; doi:10.1371/journal.pone.0019734)
Supplement: Table S1 — Inflammation, wound healing and defense response. Changes in gene expression are reported as fold-change (FC) relative to Lox mice. Significant differences between Lox and SKO were determined as described in Methods , and for both Welch's t-test and EBarrays the false discovery rate was set at 5%. All probe sets listed have a posterior probability of differential expression (PP of DE) >0.639 (soft threshold) based upon analysis by EBarrays. Additionally, Welch's t-test was used to calculate q-values and those probe sets with q-values <0.05 were considered significant. (PDF) [file pone.0019734.s002.pdf]

Supplementary Table I: Inflammation, wound healing and defense response

AFFY ID	Gene Symbol	Gene Name	FC	PP of DE	q value
1438148_at	*Cxcl3*	chemokine (C-X-C motif) ligand 3	**30.63**	1.00	0.11
1450826_a_at	*Saa3*	serum amyloid A 3	**25.47**	1.00	0.055
1449984_at	*Cxcl2*	chemokine (C-X-C motif) ligand 2	**22.85**	1.00	0.075
1448377_at	*Slpi*	secretory leukocyte peptidase inhibitor	**21.69**	1.00	0.057
1448756_at	*S100a9*	S100 calcium binding protein A9 (calgranulin B)	**20.77**	1.00	0.058
1450788_at	*Saa1*	serum amyloid A 1	**14.12**	1.00	0.055
1419394_s_at	*S100a8*	S100 calcium binding protein A8 (calgranulin A)	**11.98**	1.00	0.055
1419728_at	*Cxcl5*	chemokine (C-X-C motif) ligand 5	**8.98**	1.00	0.102
1418609_at	*Il1f6*	interleukin 1 family, member 6	**7.85**	1.00	0.082
1419209_at	*Cxcl1*	chemokine (C-X-C motif) ligand 1	**7.71**	1.00	0.089
1421688_a_at	*Ccl1*	chemokine (C-C motif) ligand 1	**6.36**	1.00	0.061
1419492_s_at	*Defb1*	defensin beta 1	**5.40**	1.00	0.06
1422029_at	*Ccl20*	chemokine (C-C motif) ligand 20	**5.00**	1.00	0.089
1419491_at	*Defb1*	defensin beta 1	**4.68**	1.00	0.062
1419600_at	*Defb4*	defensin beta 4	**4.66**	1.00	0.053
1426300_at	*Alcam*	activated leukocyte cell adhesion molecule	**4.35**	1.00	0.044
1419561_at	*Ccl3*	chemokine (C-C motif) ligand 3	**4.10**	1.00	0.122
1417932_at	*Il18*	interleukin 18	**4.04**	1.00	0.039
1421806_at	*Defb3*	defensin beta 3	**3.97**	1.00	0.031
1449399_a_at	*Il1b*	interleukin 1 beta	**3.89**	1.00	0.082
1434376_at	*Cd44*	CD44 antigen	**3.73**	1.00	0.06
1452483_a_at	*Cd44*	CD44 antigen	**3.68**	1.00	0.071
1423760_at	*Cd44*	CD44 antigen	**3.65**	1.00	0.099
1448823_at	*Cxcl12*	chemokine (C-X-C motif) ligand 12	**3.48**	1.00	0.068
1417925_at	*Ccl22*	chemokine (C-C motif) ligand 22	**3.28**	1.00	0.052
1437467_at	*Alcam*	activated leukocyte cell adhesion molecule	**3.26**	1.00	0.058
1417574_at	*Cxcl12*	chemokine (C-X-C motif) ligand 12	**3.20**	1.00	0.039
1418457_at	*Cxcl14*	chemokine (C-X-C motif) ligand 14	**3.11**	1.00	0.113
1449195_s_at	*Cxcl16*	chemokine (C-X-C motif) ligand 16	**2.99**	1.00	0.062
1417483_at	*Nfkbiz*	nuclear factor of kappa light polypeptide gene enhancer in B-cells inhibitor, zeta	**2.87**	1.00	0.082
1437466_at	*Alcam*	activated leukocyte cell adhesion molecule	**2.84**	1.00	0.04
1418718_at	*Cxcl16*	chemokine (C-X-C motif) ligand 16	**2.78**	1.00	0.067
1451798_at	*Il1rn*	interleukin 1 receptor antagonist	**2.77**	1.00	0.051
1426301_at	*Alcam*	activated leukocyte cell adhesion molecule	**2.74**	1.00	0.073
1423466_at	*Ccr7*	chemokine (C-C motif) receptor 7	**2.72**	1.00	0.069
1448710_at	*Cxcr4*	chemokine (C-X-C motif) receptor 4	**2.69**	1.00	0.129
1419609_at	*Ccr1*	chemokine (C-C motif) receptor 1	**2.58**	0.99	0.175
1418456_a_at	*Cxcl14*	chemokine (C-X-C motif) ligand 14	**2.48**	1.00	0.054
1421807_at	*Defb6*	defensin beta 6	**2.41**	1.00	0.071
1425715_at	*Il1f8*	interleukin 1 family, member 8	**2.34**	1.00	0.148
1433699_at	*Tnfaip3*	tumor necrosis factor, alpha-induced protein 3	**2.28**	1.00	0.037
1457644_s_at	*Cxcl1*	chemokine (C-X-C motif) ligand 1	**2.21**	1.00	0.104
1418424_at	*Tnfaip6*	tumor necrosis factor alpha induced protein 6	**2.19**	1.00	0.127
1419413_at	*Ccl17*	chemokine (C-C motif) ligand 17	**2.16**	1.00	0.077
1425663_at	*Il1rn*	interleukin 1 receptor antagonist	**2.12**	1.00	0.095
1421370_a_at	*Il1f5*	interleukin 1 family, member 5 (delta)	**2.04**	1.00	0.109
1458299_s_at	*Nfkbie*	nuclear factor of kappa light polypeptide gene enhancer in B-cells inhibitor, epsilon	**1.99**	1.00	0.137
1416200_at	*Il33*	interleukin 33	**1.97**	1.00	0.11
1436074_at	*Nfkbid*	nuclear factor of kappa light polypeptide gene enhancer in B-cells inhibitor, delta	**1.93**	0.94	0.142
1421186_at	*Ccr2*	chemokine (C-C motif) receptor 2	**1.92**	0.96	0.148
1420380_at	*Ccl2*	chemokine (C-C motif) ligand 2	**1.89**	0.93	0.145
1424727_at	*Ccr5*	chemokine (C-C motif) receptor 5	**1.86**	0.95	0.167
1449326_x_at	*Saa2*	serum amyloid A 2	**1.84**	1.00	0.062
1420653_at	*Tgfb1*	transforming growth factor, beta 1	**1.81**	0.98	0.149
1431843_a_at	*Nfkbie*	nuclear factor of kappa light polypeptide gene enhancer in B-cells inhibitor, epsilon	**1.77**	1.00	0.134
1448728_a_at	*Nfkbiz*	nuclear factor of kappa light polypeptide gene enhancer in B-cells inhibitor, zeta	**1.64**	0.98	0.118
1419188_s_at	*Ccl27a*	chemokine (C-C motif) ligand 27A	**1.64**	1.00	0.05
1423017_a_at	*Il1rn*	interleukin 1 receptor antagonist	**1.62**	0.74	0.205
1425902_a_at	*Nfkb2*	nuclear factor of kappa light polypeptide gene enhancer in B-cells 2, p49/p100	**1.61**	0.94	0.168
1425958_at	*Il1f9*	interleukin 1 family, member 9	**1.61**	0.90	0.152
1457404_at	*Nfkbiz*	nuclear factor of kappa light polypeptide gene enhancer in B-cells inhibitor, zeta	**1.60**	1.00	0.104
1427705_a_at	*Nfkb1*	nuclear factor of kappa light polypeptide gene enhancer in B-cells 1, p105	**1.56**	1.00	0.074
1417865_at	*Tnfaip1*	tumor necrosis factor, alpha-induced protein 1 (endothelial)	**1.53**	0.90	0.166
1426507_at	*Il1f5*	interleukin 1 family, member 5 (delta)	**1.52**	0.99	0.092
1448950_at	*Il1r1*	interleukin 1 receptor, type I	**1.52**	1.00	0.071
1419607_at	*Tnf*	tumor necrosis factor	**1.49**	0.94	0.122
1418777_at	*Ccl25*	chemokine (C-C motif) ligand 25	**1.49**	0.93	0.114
1421628_at	*Il18r1*	interleukin 18 receptor 1	**1.47**	0.90	0.151
1460351_at	*S100a11*	S100 calcium binding protein A11 (calgizzarin)	**1.47**	0.98	0.042
1438855_x_at	*Tnfaip2*	tumor necrosis factor, alpha-induced protein 2	**1.44**	0.66	0.142
1427419_x_at	*Ccr9*	chemokine (C-C motif) receptor 9	**1.39**	0.84	0.101
1449585_at	*Il1rap*	interleukin 1 receptor accessory protein	**1.37**	0.95	0.078
1425860_x_at	*Cklf*	chemokine-like factor	**1.36**	0.88	0.101
1424495_a_at	*Cklf*	chemokine-like factor	**1.35**	0.69	0.138
1436242_a_at	*Cklf*	chemokine-like factor	**1.34**	0.91	0.086
1449951_at	*Nfkbil1*	nuclear factor of kappa light polypeptide gene enhancer in B-cells inhibitor-like 1	**0.69**	0.81	0.125
1422112_at	*Ccbp2*	chemokine binding protein 2	**0.69**	0.99	0.115
1439348_at	*S100a10*	S100 calcium binding protein A10 (calpactin)	**0.61**	1.00	0.085
1426139_a_at	*Ccrl1*	chemokine (C-C motif) receptor-like 1	**0.57**	1.00	0.102
1451957_at	*Il1f10*	interleukin 1 family, member 10	**0.55**	1.00	0.129
1425155_x_at	*Csf1*	colony stimulating factor 1 (macrophage)	**0.52**	1.00	0.093
1437937_at	*Ccbp2*	chemokine binding protein 2	**0.50**	1.00	0.116
1417625_s_at	*Cxcr7*	chemokine (C-X-C motif) receptor 7	**0.42**	1.00	0.071
1450488_at	*Ccl24*	chemokine (C-C motif) ligand 24	**0.29**	1.00	0.106
1419532_at	*Il1r2*	interleukin 1 receptor, type II	**0.28**	1.00	0.054
1427778_at	*Defb8*	defensin beta 8	**0.20**	1.00	0.146

See article file for table legend
