# Supplementary material for: Metabolic Changes in Skin Caused by Scd1 Deficiency: A Focus on Retinol Metabolism
Source: PLoS One. 2011 May 9;6(5):e19734. doi: 10.1371/journal.pone.0019734 (PMC3090422; doi:10.1371/journal.pone.0019734)
Supplement: Table S2 — Miscellaneous categories. Changes in gene expression are reported as fold-change (FC) relative to Lox mice. Significant differences between Lox and SKO were determined as described in Methods , and for both Welch's t-test and EBarrays the false discovery rate was set at 5%. All probe sets listed have a posterior probability of differential expression (PP of DE) >0.639 (soft threshold) based upon analysis by EBarrays. Additionally, Welch's t-test was used to calculate q-values and those probe sets with q-values <0.05 were considered significant. (PDF) [file pone.0019734.s003.pdf]

Supplementary Table II: Miscellaneous categories

Lipoxygenases

AFFY ID	Gene Symbol	Gene Name	FC	PP of DE	q value
1426039_a_at	*Alox12e*	arachidonate lipoxygenase, epidermal	**3.123**	1	0.023
1420338_at	*Alox15*	arachidonate 15-lipoxygenase	**2.509**	1	0.066
1441962_at	*Alox5*	arachidonate 5-lipoxygenase	**0.647**	0.978	0.112
1422699_at	*Alox12*	arachidonate 12-lipoxygenase	**0.562**	1	0.05

Fatty acid oxidation

AFFY ID	Gene Symbol	Gene Name	FC	PP of DE	q value
1450966_at	*Crot*	carnitine O-octanoyltransferase	**2.906**	1	0.085
1460409_at	*Cpt1a*	carnitine palmitoyltransferase 1a, liver	**2.803**	1	0.063
1438156_x_at	*Cpt1a*	carnitine palmitoyltransferase 1a, liver	**2.07**	1	0.031
1434866_x_at	*Cpt1a*	carnitine palmitoyltransferase 1a, liver	**2.018**	1	0.078
1416409_at	*Acox1*	acyl-Coenzyme A oxidase 1, palmitoyl	**1.817**	1	0.064

Circadian Rhythm

AFFY ID	Gene Symbol	Gene Name	FC	PP of DE	q value
1458176_at	*Per3*	period homolog 3 (Drosophila)	**0.594**	0.987	0.111
1441445_at	*Per3*	period homolog 3 (Drosophila)	**0.542**	0.752	0.176
1434735_at	*Hlf*	hepatic leukemia factor	**0.527**	0.987	0.126
1417602_at	*Per2*	period homolog 2 (Drosophila)	**0.447**	0.921	0.18
1424175_at	*Tef*	thyrotroph embryonic factor	**0.431**	1	0.077
1442243_at	*Per3*	period homolog 3 (Drosophila)	**0.406**	1	0.057
1457350_at	*Per2*	period homolog 2 (Drosophila)	**0.384**	1	0.08
1426383_at	*Cry2*	cryptochrome 2 (photolyase-like)	**0.336**	1	0.036
1438211_s_at	*Dbp*	D site albumin promoter binding protein	**0.324**	1	0.067
1418174_at	*Dbp*	D site albumin promoter binding protein	**0.271**	1	0.068

Glucose metabolism

AFFY ID	Gene Symbol	Gene Name	FC	PP of DE	q value
1426600_at	*Slc2a1*	solute carrier family 2 (facilitated glucose transporter), member 1	**3.505**	1	0.068
1426599_a_at	*Slc2a1*	solute carrier family 2 (facilitated glucose transporter), member 1	**3.113**	1	0.082
1434773_a_at	*Slc2a1*	solute carrier family 2 (facilitated glucose transporter), member 1	**2.656**	1	0.048
1416069_at	*Pfkp*	phosphofructokinase, platelet	**2.493**	1	0.045
1439148_a_at	*Pfkl*	phosphofructokinase, liver, B-type	**1.488**	0.998	0.072
1450269_a_at	*Pfkl*	phosphofructokinase, liver, B-type	**1.439**	0.968	0.1
AFFX-PheX-3_at	*Pcx*	pyruvate carboxylase	**0.693**	0.99	0.069
AFFX-PheX-M_at	*Pcx*	pyruvate carboxylase	**0.665**	0.752	0.117
1419146_a_at	*Gck*	glucokinase	**0.643**	0.981	0.144
1450196_s_at	*Gys1*	glycogen synthase 1, muscle	**0.521**	1	0.115
1433504_at	*Pygb*	brain glycogen phosphorylase	**0.521**	1	0.062
1448825_at	*Pdk2*	pyruvate dehydrogenase kinase, isoenzyme 2	**0.456**	1	0.067
AFFX-PyruCarbM	*Pcx*	pyruvate carboxylase	**0.437**	0.999	0.095
1416737_at	*Gys1*	glycogen synthase 1, muscle	**0.42**	1	0.03
1427213_at	*Pfkfb1*	6-phosphofructo-2-kinase/fructose-2,6-biphosphatase 1	**0.409**	1	0.03
1448429_at	*Gyg*	glycogenin	**0.403**	1	0.053
1416383_a_at	*Pcx*	pyruvate carboxylase	**0.396**	0.998	0.115
1449088_at	*Fbp2*	fructose bisphosphatase 2	**0.363**	1	0.097
1425303_at	*Gck*	glucokinase	**0.359**	1	0.073
1415959_at	*Slc2a4*	solute carrier family 2 (facilitated glucose transporter), member 4	**0.354**	1	0.139
1459522_s_at	*Gyg*	glycogenin	**0.338**	1	0.053
1417273_at	*Pdk4*	pyruvate dehydrogenase kinase, isoenzyme 4	**0.312**	1	0.065
1415958_at	*Slc2a4*	solute carrier family 2 (facilitated glucose transporter), member 4	**0.266**	1	0.056
1448602_at	*Pygm*	muscle glycogen phosphorylase	**0.262**	1	0.051
1416780_at	*Pfkm*	phosphofructokinase, muscle	**0.256**	1	0.074

Oxidative stress and free radical production

AFFY ID	Gene Symbol	Gene Name	FC	PP of DE	q value
1449279_at	*Gpx2*	glutathione peroxidase 2	**24.504**	1	0.043
1444566_at	*Ucp2*	uncoupling protein 2 (mitochondrial, proton carrier)	**8.596**	1	0.04
1447046_at	*Ucp2*	uncoupling protein 2 (mitochondrial, proton carrier)	**5.898**	1	0.052
1448188_at	*Ucp2*	uncoupling protein 2 (mitochondrial, proton carrier)	**4.088**	1	0.083
1459741_x_at	*Ucp2*	uncoupling protein 2 (mitochondrial, proton carrier)	**3.881**	1	0.078
1459740_s_at	*Ucp2*	uncoupling protein 2 (mitochondrial, proton carrier)	**3.586**	1	0.079
1420657_at	*Ucp3*	uncoupling protein 3 (mitochondrial, proton carrier)	**0.647**	0.996	0.132
1417836_at	*Gpx7*	glutathione peroxidase 7	**0.577**	0.998	0.122
1451124_at	*Sod1*	superoxide dismutase 1, soluble	**0.55**	1	0.023
1459976_s_at	*Sod1*	superoxide dismutase 1, soluble	**0.55**	1	0.045
1449106_at	*Gpx3*	glutathione peroxidase 3	**0.468**	1	0.054

Miscellaneous energy metabolism

AFFY ID	Gene Symbol	Gene Name	FC	PP of DE	q value
1419127_at	*Npy*	neuropeptide Y	**33.085**	1	0.032
1418937_at	*Dio2*	deiodinase, iodothyronine, type II	**5.34**	1	0.08
1421690_s_at	*Agrp*	agouti related protein	**1.643**	0.812	0.162

Iron Metabolism

AFFY ID	Gene Symbol	Gene Name	FC	PP of DE	q value
1417061_at	*Slc40a1*	solute carrier family 40 (iron-regulated transporter), member 1	**5.211**	1	0.058
1425381_a_at	*Trfr2*	transferrin receptor 2	**0.542**	0.989	0.123
1459994_x_at	*Trfr2*	transferrin receptor 2	**0.495**	0.926	0.153
1429223_a_at	*Hfe2*	hemochromatosis type 2 (juvenile) (human homolog)	**0.235**	1	0.05
1419197_x_at	*Hamp*	hepcidin antimicrobial peptide	**0.141**	1	0.037
1419196_at	*Hamp*	hepcidin antimicrobial peptide	**0.133**	1	0.054
1436643_x_at	*Hamp2*	hepcidin antimicrobial peptide 2	**0.032**	1	0.051

Aquaporins

AFFY ID	Gene Symbol	Gene Name	FC	PP of DE	q value
1424011_at	*Aqp9*	aquaporin 9	**3.326**	1	0.045
1422007_at	*Aqp3*	aquaporin 3	**2.235**	0.987	0.142
1422008_a_at	*Aqp3*	aquaporin 3	**2.001**	0.743	0.208
1450460_at	*Aqp3*	aquaporin 3	**1.822**	0.789	0.184
1421605_a_at	*Aqp9*	aquaporin 9	**1.581**	1	0.059

See article file for table legend
