# Supplementary material for: Metabolic Changes in Skin Caused by Scd1 Deficiency: A Focus on Retinol Metabolism
Source: PLoS One. 2011 May 9;6(5):e19734. doi: 10.1371/journal.pone.0019734 (PMC3090422; doi:10.1371/journal.pone.0019734)
Supplement: Table S3 — Proteases and peptidases. Changes in gene expression are reported as fold-change (FC) relative to Lox mice. Significant differences between Lox and SKO were determined as described in Methods , and for both Welch's t-test and EBarrays the false discovery rate was set at 5%. All probe sets listed have a posterior probability of differential expression (PP of DE) >0.639 (soft threshold) based upon analysis by EBarrays. Additionally, Welch's t-test was used to calculate q-values and those probe sets with q-values <0.05 were considered significant. (PDF) [file pone.0019734.s004.pdf]

Supplementary Table III: Proteases and peptidases

AFFY ID	Gene Symbol	Gene Name	FC	PP of DE	q value
1417256_at	*Mmp13*	matrix metallopeptidase 13	**71.219**	1	0.056
1449153_at	*Mmp12*	matrix metallopeptidase 12	**67.726**	1	0.056
1448982_at	*Klk6*	kallikrein related-peptidase 6	**38.967**	1	0.067
1440686_at	*LOC100048538 /// Prss27*	similar to protease, serine 27 /// protease, serine 27	**35.228**	1	0.03
1460227_at	*Timp1*	tissue inhibitor of metalloproteinase 1	**8.436**	1	0.065
1417234_at	*Mmp11*	matrix metallopeptidase 11	**7.424**	1	0.058
1421977_at	*Mmp19*	matrix metallopeptidase 19	**4.335**	1	0.077
1416298_at	*Mmp9*	matrix metallopeptidase 9	**4.322**	1	0.064
1457724_at	*Ctsl*	cathepsin L	**4.191**	1	0.03
1451310_a_at	*Ctsl*	cathepsin L	**3.249**	1	0.065
1448732_at	*Ctsb*	cathepsin B	**2.958**	1	0.086
1417492_at	*Ctsb*	cathepsin B	**2.929**	1	0.085
1416572_at	*Mmp14*	matrix metallopeptidase 14 (membrane-inserted)	**2.587**	1	0.083
1448591_at	*Ctss*	cathepsin S	**2.259**	0.851	0.133
1420450_at	*Mmp10*	matrix metallopeptidase 10	**2.199**	0.984	0.133
1417491_at	*Ctsb*	cathepsin B	**2.187**	1	0.057
1421976_at	*Mmp19*	matrix metallopeptidase 19	**2.186**	0.998	0.111
1419088_at	*Timp3*	tissue inhibitor of metalloproteinase 3	**2.031**	0.987	0.125
1416382_at	*Ctsc*	cathepsin C	**1.844**	0.988	0.152
1437939_s_at	*Ctsc*	cathepsin C	**1.669**	0.955	0.147
1417490_at	*Ctsb*	cathepsin B	**1.646**	0.941	0.136
1449366_at	*Mmp8*	matrix metallopeptidase 8	**1.635**	1	0.072
1448128_at	*Ctsa*	cathepsin A	**1.625**	0.976	0.124
1449334_at	*Timp3*	tissue inhibitor of metalloproteinase 3	**1.616**	0.984	0.107
1448118_a_at	*Ctsd*	cathepsin D	**1.509**	0.994	0.055
1448598_at	*Mmp17*	matrix metallopeptidase 17	**0.727**	0.819	0.131
1433662_s_at	*Timp2*	tissue inhibitor of metalloproteinase 2	**0.682**	0.933	0.125
1437462_x_at	*Mmp15*	matrix metallopeptidase 15	**0.677**	0.868	0.127
1460287_at	*Timp2*	tissue inhibitor of metalloproteinase 2	**0.669**	0.998	0.069
1454677_at	*Timp2*	tissue inhibitor of metalloproteinase 2	**0.621**	0.944	0.082
1439364_a_at	*Mmp2*	matrix metallopeptidase 2	**0.553**	0.87	0.117
1416136_at	*Mmp2*	matrix metallopeptidase 2	**0.552**	0.984	0.093
1450652_at	*Ctsk*	cathepsin K	**0.473**	0.969	0.115
1450974_at	*Timp4*	tissue inhibitor of metalloproteinase 4	**0.396**	1	0.052
1417281_a_at	*Mmp23*	matrix metallopeptidase 23	**0.361**	1	0.055
1417282_at	*Mmp23*	matrix metallopeptidase 23	**0.346**	1	0.076
1423405_at	*Timp4*	tissue inhibitor of metalloproteinase 4	**0.267**	1	0.03

ADAMs family of metallopeptidases

AFFY ID	Gene Symbol	Gene Name	FC	PP of DE	q value
1456901_at	*Adamts20*	a disintegrin-like and metallopeptidase (reprolysin type) with thrombospondin type 1 motif, 20	**8.778**	1	0.048
1421172_at	*Adam12*	a disintegrin and metallopeptidase domain 12 (meltrin alpha)	**4.71**	1	0.094
1450716_at	*Adamts1*	a disintegrin-like and metallopeptidase (reprolysin type) with thrombospondin type 1 motif, 1	**2.992**	1	0.052
1427056_at	*Adamts15*	a disintegrin-like and metallopeptidase (reprolysin type) with thrombospondin type 1 motif, 15	**2.775**	1	0.085
1416871_at	*Adam8*	a disintegrin and metallopeptidase domain 8	**2.619**	0.989	0.129
1452595_at	*Adamts4*	a disintegrin-like and metallopeptidase (reprolysin type) with thrombospondin type 1 motif, 4	**2.572**	1	0.095
1450105_at	*Adam10*	a disintegrin and metallopeptidase domain 10	**2.499**	1	0.092
1421171_at	*Adam12*	a disintegrin and metallopeptidase domain 12 (meltrin alpha)	**2.253**	1	0.048
1455965_at	*Adamts4*	a disintegrin-like and metallopeptidase (reprolysin type) with thrombospondin type 1 motif, 4	**2.227**	1	0.056
1452339_at	*Adamts7*	a disintegrin-like and metallopeptidase (reprolysin type) with thrombospondin type 1 motif, 7	**2.006**	0.971	0.182
1437785_at	*Adamts9*	a disintegrin-like and metallopeptidase (reprolysin type) with thrombospondin type 1 motif, 9	**1.59**	0.989	0.113
1441693_at	*Adamts3*	a disintegrin-like and metallopeptidase (reprolysin type) with thrombospondin type 1 motif, 3	**1.316**	0.807	0.102
1421859_at	*Adam17*	a disintegrin and metallopeptidase domain 17	**1.303**	0.67	0.087
1443378_s_at	*Adam1a*	a disintegrin and metallopeptidase domain 1a	**0.715**	0.92	0.095
1440668_at	*Adamtsl3*	ADAMTS-like 3	**0.672**	0.995	0.1
1444628_at	*Adam33*	a disintegrin and metallopeptidase domain 33	**0.638**	0.999	0.075
1435293_at	*Adam22*	a disintegrin and metallopeptidase domain 22	**0.626**	0.996	0.092
1455720_at	*Adamts2*	a disintegrin-like and metallopeptidase (reprolysin type) with thrombospondin type 1 motif, 2	**0.587**	1	0.088
1451904_a_at	*Adam33*	a disintegrin and metallopeptidase domain 33	**0.517**	1	0.056
1457058_at	*Adamts2*	a disintegrin-like and metallopeptidase (reprolysin type) with thrombospondin type 1 motif, 2	**0.479**	1	0.073
1442063_at	*Adamtsl1*	ADAMTS-like 1	**0.429**	1	0.113
1429214_at	*Adamtsl2*	ADAMTS-like 2	**0.418**	0.998	0.152
1435990_at	*Adamts2*	a disintegrin-like and metallopeptidase (reprolysin type) with thrombospondin type 1 motif, 2	**0.402**	1	0.075
1447946_at	*Adam23*	a disintegrin and metallopeptidase domain 23	**0.361**	1	0.031
1438266_at	*Adamtsl5*	ADAMTS-like 5	**0.345**	1	0.048
1430313_at	*Adamtsl1*	ADAMTS-like 1	**0.337**	1	0.098
1451932_a_at	*Adamtsl4*	ADAMTS-like 4	**0.313**	1	0.04

See article file for table legend
