# Supplementary material for: Metabolic Changes in Skin Caused by Scd1 Deficiency: A Focus on Retinol Metabolism
Source: PLoS One. 2011 May 9;6(5):e19734. doi: 10.1371/journal.pone.0019734 (PMC3090422; doi:10.1371/journal.pone.0019734)
Supplement: Table S4 — Collagens, keratins, gap junctions and tight junctions. Changes in gene expression are reported as fold-change (FC) relative to Lox mice. Significant differences between Lox and SKO were determined as described in Methods , and for both Welch's t-test and EBarrays the false discovery rate was set at 5%. All probe sets listed have a posterior probability of differential expression (PP of DE) >0.639 (soft threshold) based upon analysis by EBarrays. Additionally, Welch's t-test was used to calculate q-values and those probe sets with q-values <0.05 were considered significant. (PDF) [file pone.0019734.s005.pdf]

Supplementary Table IV: Collagens, keratins, gap junctions and tight junctions

Collagen family

AFFY ID	Gene Symbol	Gene Name	FC	PP of DE	q value
1434411_at	*Col12a1*	collagen, type XII, alpha 1	**10.606**	1	0.042
1455627_at	*Col8a1*	collagen, type VIII, alpha 1	**4.763**	1	0.092
1427391_a_at	*Col12a1*	collagen, type XII, alpha 1	**4.291**	1	0.057
1447819_x_at	*Col8a1*	collagen, type VIII, alpha 1	**3.15**	1	0.072
1418599_at	*Col11a1*	collagen, type XI, alpha 1	**2.938**	1	0.1
1418440_at	*Col8a1*	collagen, type VIII, alpha 1	**2.897**	1	0.058
1449154_at	*Col11a1*	collagen, type XI, alpha 1	**2.632**	1	0.084
1429549_at	*Col27a1*	collagen, type XXVII, alpha 1	**2.134**	1	0.13
1418237_s_at	*Col18a1*	collagen, type XVIII, alpha 1	**2.065**	1	0.052
1426955_at	*Col18a1*	collagen, type XVIII, alpha 1	**1.997**	1	0.084
1422253_at	*Col10a1*	collagen, type X, alpha 1	**1.827**	0.935	0.175
1419613_at	*Col7a1*	collagen, type VII, alpha 1	**1.651**	0.995	0.094
1434667_at	*Col8a2*	collagen, type VIII, alpha 2	**1.539**	0.794	0.145
1420383_a_at	*Col4a3bp*	collagen, type IV, alpha 3 (Goodpasture antigen) binding protein	**1.407**	0.86	0.138
1425235_s_at	*Col20a1*	collagen, type XX, alpha 1	**0.748**	0.882	0.094
1440911_at	*Col23a1*	collagen, type XXIII, alpha 1	**0.732**	0.791	0.139
1430855_at	*Col20a1*	collagen, type XX, alpha 1	**0.718**	0.809	0.13
1438779_at	*Col4a3*	collagen, type IV, alpha 3	**0.689**	0.801	0.144
1425236_at	*Col20a1*	collagen, type XX, alpha 1	**0.684**	1	0.046
1429280_at	*Col22a1*	collagen, type XXII, alpha 1	**0.648**	0.906	0.152
1425772_at	*Col4a4*	collagen, type IV, alpha 4	**0.64**	1	0.04
1429209_at	*Col23a1*	collagen, type XXIII, alpha 1	**0.575**	1	0.081
1425234_at	*Col20a1*	collagen, type XX, alpha 1	**0.558**	0.984	0.12
1453084_s_at	*Col22a1*	collagen, type XXII, alpha 1	**0.441**	1	0.094
1423669_at	*Col1a1*	collagen, type I, alpha 1	**0.354**	0.997	0.103
1445328_at	*Col4a4*	collagen, type IV, alpha 4	**0.313**	1	0.102
1440250_at	*Col4a4*	collagen, type IV, alpha 4	**0.287**	1	0.1
1446326_at	*Col1a2*	collagen, type I, alpha 2	**0.253**	1	0.066

Keratin family

AFFY ID	Gene Symbol	Gene Name	FC	PP of DE	q value
1430208_at	*Krt42*	keratin 42	**8.774**	1	0.036
1423952_a_at	*Krt7*	keratin 7	**8.439**	1	0.037
1439765_x_at	*Krt42*	keratin 42	**3.85**	1	0.06
1423227_at	*Krt17*	keratin 17	**2.878**	1	0.03
1455573_at	*Krt14*	keratin 14	**2.814**	1	0.051
1419619_at	*Krt80*	keratin 80	**2.507**	1	0.037
1449378_at	*Krt27*	keratin 27	**2.506**	0.994	0.17
1427352_at	*Krt79*	keratin 79	**2.454**	1	0.062
1436557_at	*Krt73*	keratin 73	**2.071**	1	0.079
1423691_x_at	*Krt8*	keratin 8	**2.01**	1	0.085
1448169_at	*Krt18*	keratin 18	**1.83**	0.999	0.093
1460347_at	*Krt14*	keratin 14	**1.714**	1	0.07
1448457_at	*Krt71*	keratin 71	**1.712**	0.938	0.18
1435989_x_at	*Krt8*	keratin 8	**1.687**	0.993	0.145
1420647_a_at	*Krt8*	keratin 8	**1.683**	0.999	0.114
1423935_x_at	*Krt14*	keratin 14	**1.455**	0.879	0.085
1437344_x_at	*Krt13*	keratin 13	**0.74**	0.922	0.059
1438394_x_at	*Krt4*	keratin 4	**0.689**	0.999	0.047
1438849_at	*Krt78*	keratin 78	**0.651**	0.993	0.126
1430132_at	*Krt28*	keratin 28	**0.638**	0.973	0.108
1417156_at	*Krt19*	keratin 19	**0.457**	1	0.096
1422667_at	*Krt15*	keratin 15	**0.253**	1	0.06
1420728_at	*Krt32*	keratin 32	**0.184**	1	0.03
1453327_at	*Krt24*	keratin 24	**0.145**	1	0.046
1427154_at	*Krt2*	keratin 2	**0.112**	1	0.11
1427378_at	*Krt75*	keratin 75	**0.11**	1	0.057

Gap junction and tight junction formation

AFFY ID	Gene Symbol	Gene Name	FC	PP of DE	q value
1423271_at	*Gjb2*	gap junction protein, beta 2	**38.174**	1	0.045
1418748_at	*Casp14*	caspase 14	**9.085**	1	0.039
1426911_at	*Dsc2*	desmocollin 2	**5.461**	1	0.07
1448397_at	*Gjb6*	gap junction protein, beta 6	**4.097**	1	0.149
1415800_at	*Gja1*	gap junction protein, alpha 1	**3.912**	1	0.073
1421156_a_at	*Dsc2*	desmocollin 2	**3.831**	1	0.099
1449740_s_at	*Dsg2*	desmoglein 2	**3.133**	1	0.06
1418283_at	*Cldn4*	claudin 4	**3.109**	1	0.085
1425619_s_at	*Dsg2*	desmoglein 2	**3.095**	1	0.058
1434600_at	*Tjp2*	tight junction protein 2	**3.089**	1	0.037
1439476_at	*Dsg2*	desmoglein 2	**3.03**	1	0.066
1434599_a_at	*Tjp2*	tight junction protein 2	**3.009**	1	0.051
1450984_at	*Tjp2*	tight junction protein 2	**2.847**	1	0.042
1450014_at	*Cldn1*	claudin 1	**2.825**	1	0.068
1450985_a_at	*Tjp2*	tight junction protein 2	**2.728**	1	0.061
1438945_x_at	*Gja1*	gap junction protein, alpha 1	**2.697**	1	0.094
1437932_a_at	*Cldn1*	claudin 1	**2.637**	1	0.084
1422179_at	*Gjb4*	gap junction protein, beta 4	**2.608**	1	0.057
1426673_at	*Cdh3*	cadherin 3	**2.596**	1	0.05
1437992_x_at	*Gja1*	gap junction protein, alpha 1	**2.59**	1	0.088
1448873_at	*Ocln*	occludin	**2.495**	1	0.054
1438650_x_at	*Gja1*	gap junction protein, alpha 1	**2.277**	1	0.113
1415801_at	*Gja1*	gap junction protein, alpha 1	**2.18**	1	0.088
1438973_x_at	*Gja1*	gap junction protein, alpha 1	**2.056**	0.999	0.099
1435191_at	*Cdsn*	corneodesmosin	**1.988**	1	0.069
1448261_at	*Cdh1*	cadherin 1	**1.985**	1	0.079
1449741_at	*Dsg2*	desmoglein 2	**1.849**	1	0.067
1417749_a_at	*Tjp1*	tight junction protein 1	**1.642**	0.975	0.127
1416715_at	*Gjb3*	gap junction protein, beta 3	**1.569**	0.898	0.106
1441255_at	*Cdh3*	cadherin 3	**1.414**	0.97	0.085
1455519_at	*Dsg1b*	desmoglein 1 beta	**1.369**	0.737	0.123
1422274_at	*Gja8*	gap junction protein, alpha 8	**0.754**	0.84	0.099
1423551_at	*Cdh13*	cadherin 13	**0.687**	0.765	0.115
1420345_at	*Cldn14*	claudin 14	**0.645**	0.999	0.076
1434115_at	*Cdh13*	cadherin 13	**0.635**	1	0.065
1439427_at	*Cldn9*	claudin 9	**0.606**	1	0.059
1416255_at	*Gja4*	gap junction protein, alpha 4	**0.56**	0.999	0.106
1426332_a_at	*Cldn3*	claudin 3	**0.535**	0.999	0.123
1430237_at	*Cldn22*	claudin 22	**0.53**	1	0.061
1448393_at	*Cldn7*	claudin 7	**0.523**	1	0.065
1417896_at	*Tjp3*	tight junction protein 3	**0.514**	1	0.105
1418093_a_at	*Egf*	epidermal growth factor	**0.484**	1	0.05
1434651_a_at	*Cldn3*	claudin 3	**0.481**	1	0.121
1460569_x_at	*Cldn3*	claudin 3	**0.473**	1	0.103
1451701_x_at	*Cldn3*	claudin 3	**0.457**	1	0.118
1449204_at	*Gjb5*	gap junction protein, beta 5	**0.404**	1	0.052
1417839_at	*Cldn5*	claudin 5	**0.402**	1	0.048
1426147_s_at	*Cldn10*	claudin 10	**0.187**	1	0.084
